# Dynamics of the Fouling Layer Microbial Community in a Membrane Bioreactor

**DOI:** 10.1371/journal.pone.0158811

**Published:** 2016-07-11

**Authors:** Anja S. Ziegler, Simon J. McIlroy, Poul Larsen, Mads Albertsen, Aviaja A. Hansen, Nicolas Heinen, Per Halkjær Nielsen

**Affiliations:** 1 Center for Microbial Communities, Department of Chemistry and Bioscience, Aalborg University, Aalborg, Denmark; 2 Alfa Laval Nakskov A/S, Nakskov, Denmark; University of Notre Dame, UNITED STATES

## Abstract

Membrane fouling presents the greatest challenge to the application of membrane bioreactor (MBR) technology. Formation of biofilms on the membrane surface is the suggested cause, yet little is known of the composition or dynamics of the microbial community responsible. To gain an insight into this important question, we applied 16S rRNA gene amplicon sequencing with a curated taxonomy and fluorescent *in situ* hybridization to monitor the community of a pilot-scale MBR carrying out enhanced biological nitrogen and phosphorus removal with municipal wastewater. In order to track the dynamics of the fouling process, we concurrently investigated the communities of the biofilm, MBR bulk sludge, and the conventional activated sludge system used to seed the MBR system over several weeks from start-up. As the biofilm matured the initially abundant betaproteobacterial genera *Limnohabitans*, *Hydrogenophaga* and *Malikia* were succeeded by filamentous Chloroflexi and *Gordonia* as the abundant species. This study indicates that, although putative pioneer species appear, the biofilm became increasingly similar to the bulk community with time. This suggests that the microbial population in bulk water will largely determine the community structure of the mature biofilm.

## Introduction

The application of membrane bioreactors (MBRs) for wastewater treatment has grown worldwide, both in number and capacity. The MBR technology offers a range of advantages over the conventional activated sludge (CAS) process, such as decreased footprint, reduced excess sludge, higher effluent quality and operation under higher biomass concentrations. However, MBRs are faced with the disadvantage of membrane fouling, which affects the filtration efficiency over time, leading to flux decrease, increased energy consumption, and costly cleaning procedures or membrane replacement, resulting in downtime [1,2].

The fouling layer, also termed a biofilm or a gel layer, consists of adsorbed extracellular polymeric substances (EPS), as well as adsorbed or growing microorganisms of an unknown ratio, and may contribute to fouling in the long term [3,4]. The EPS components may originate from bulk water or *in situ* excretion by the bacteria growing in the biofilm. Bacteria produce a wide range of species-specific EPS, which can vary according to their environment and surroundings. Previous studies have shown that bacteria have very different adhesion characteristics and adhere to sludge flocs at different strengths [5,6], likely due to differences in their EPS production and properties. It is therefore likely that the composition and properties of the EPS layer in membrane fouling will be determined, at least partly, by the bacterial species responsible for the biofilm formation.

Few studies have investigated the identity of the microbial species in MBR fouling layers. In laboratory experiments using synthetic wastewater, the general findings are that the microbial community of the bulk sludge and the fouling layers are different, with specific bacteria preferentially growing on the membrane surface environment. A few studies have suggested the participation of members of the Proteobacteria in membrane fouling [7,8], however, only one of these was conducted using a full-scale system treating real wastewater. In addition, due to the incomplete taxonomic annotation of commonly applied databases, previous studies have in general discussed community dynamics at the phylum and class levels. Such observations are of questionable value given the phenotypic diversity encompassed by the higher-level phylogenetic groupings. The recent publication of the online MiDAS database addresses this problem with a curated taxonomy for the abundant organisms of activated sludge systems, which allows for taxonomic assignment down to genus level for most organisms present [9]. Furthermore, application of modern methods such as 16S rRNA gene sequencing [10,11] allows for high throughput sequencing of the entire community (giving 10,000–100,000 sequences per sample), providing details of the dynamics of most potentially relevant microbes present. Such methods are ideal for detailed analyses of the dynamics of the communities associated with biofouling of membranes.

In the present study, we wanted to test the hypothesis that the bacterial communities of the biofilm were different from bulk sludge in a pilot-scale MBR carrying out biological nitrogen removal and enhanced biological phosphorus removal (EBPR) with municipal wastewater, and whether a succession in the community could be observed over the development of a mature biofilm. The bacteria were identified using high throughput 16S rRNA gene amplicon sequencing and a curated MiDAS taxonomy that allows for more general comparative studies of the microbial community structure [9]. Key results were verified using a DNA extraction and amplification independent microscopy-based method, quantitative fluorescence *in situ* hybridization (qFISH).

## Materials & Methods

### MBR system and operating conditions

**Fig 1:**
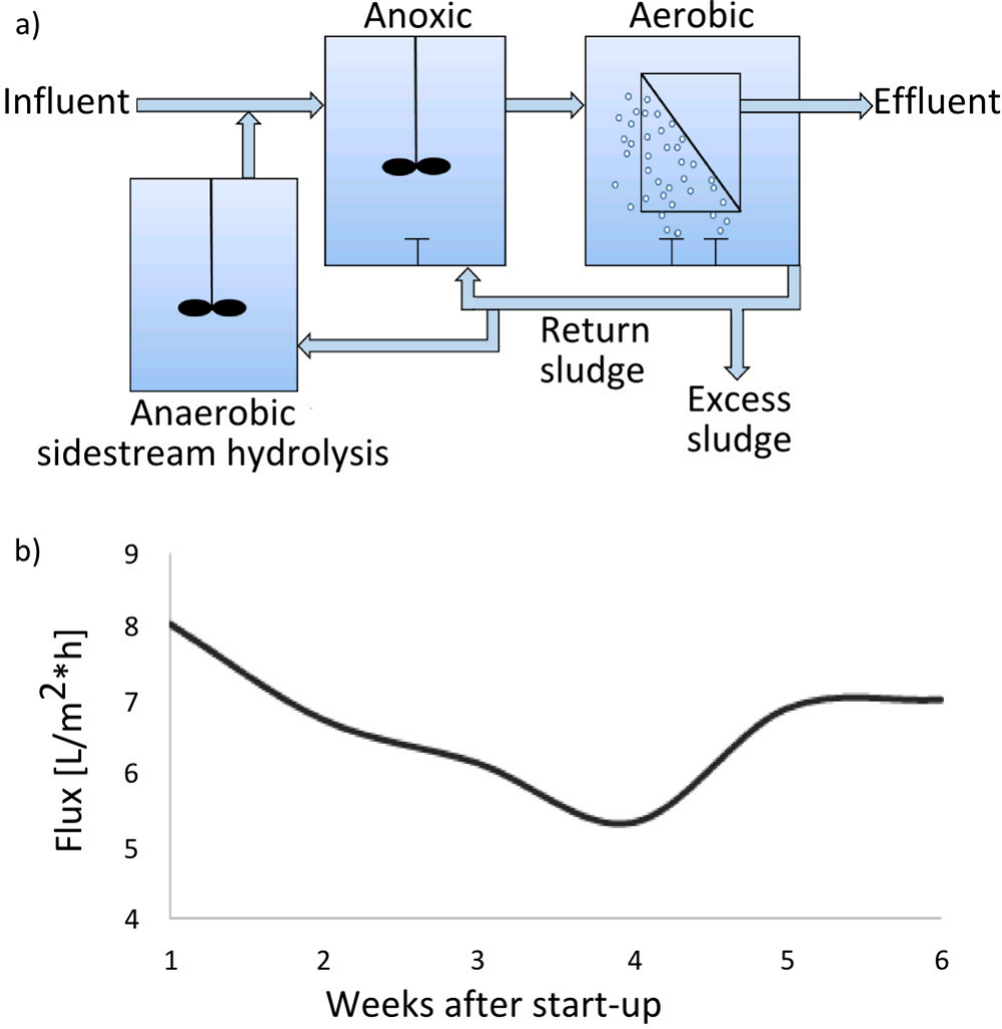
a) Schematic of the pilot scale MBR used in this study. Influent was raw wastewater and effluent was treated wastewater. b) Changes in flux (permeate flow) during the seven-week sampling period. During week 4 there was a break-down of the aerator.

The study was carried out in a pilot-scale MBR performing nitrogen removal and EBPR at the conventional full-scale plant at Aalborg West, Denmark, also with nitrogen removal and EBPR (57.049422° N, 9.864735° E). The influent wastewater came from the same primary settling tank as the full-scale plant, entering an anoxic/denitrification (2 m^3^) tank and going to an aerobic/nitrification (2 m^3^) tank through gravitation (Fig 1A). The influent flow was 0.5 m^3^/h, and it was operated with a mixed liquid suspended solids (MLSS) concentration of 3.6–5.4 kg/m^3^. An anaerobic tank (1.8 m^3^) was used for return sludge sidestream hydrolysis (RSS) (Fig 1A) to provide easily degradable substrate for EBPR and denitrification. The hydraulic retention time (HRT) in the anaerobic tank was 24 h, and the sludge retention time (SRT) 15 days. The RSS configuration is also present in the full-scale CAS plant and this process configuration is very common in Danish EBPR plants [12]. Treated water was recirculated into the system. Mixed liquid from the aeration tank was recirculated to the anaerobic tank (0.075 m^3^/h) for RSS and to the anoxic tank (1 m^3^/h). In the aerobic tank, a membrane cassette with twenty flat-sheet MFP2 membranes (polypropylene, Alfa Laval) (surface area 40 m^2^ and average pore size 0.2 μm) was submerged. A mini-membrane cassette with seven membranes (surface area 0.225 m^2^) was used for sampling and was coupled to the large membrane cassette to ensure identical transmembrane pressures (TMP). A constant TMP of 0.003 MPa (or 30 mbar) was maintained across the membrane module, and filtration was carried out intermittently (cycles of 8 min filtration and 2 min relaxation). The reactor flow was constant at 0.69–0.71 m^3^/h. The membrane modules were scoured from beneath, where bubble diffusers were positioned to supply oxygen to the mixed liquid and to clean the surface of the membranes. A compressor was used to control the airflow rate. The TMP, dissolved oxygen concentration and permeate flux were monitored online.

The membrane bioreactor was run without the sampling cassette installed for six months prior to the sampling period of this study. This period was used to achieve a stable system with regard to permeate flux, nutrient removal, and, presumably, community structure. The start-up biomass was activated sludge from the full-scale Aalborg West plant. After the six-month period, chemical cleaning of the large membrane module was carried out. New flat sheet MFP2 membranes were installed in the small membrane cassette, and the reactor was operated for seven weeks without chemical cleaning.

### Sampling

Sampling was carried out once a week for seven weeks (Oct. 2012 –Nov. 2012) in the conventional full-scale plant (CAS), pilot-scale MBR (MBR), and membrane biofilm (BF), starting at day 0 after cleaning of the MBR sheets by 1000 ppm chlorine (Cl_2_). For the liquid samples, 50 mL were collected. For the biofilm, one mini membrane was removed and replaced during the relaxation period. The fouled membrane was flushed with permeate in order to remove any foam as well as lightly bound material. Pieces of 1 cm^2^ were cut out and processed for microscopic analysis. The remaining fouling material was scraped off the membrane into 5 mL permeate using a cell scraper (Sarstedt, Germany, no. 83.1832). For both the liquid and the biofilm samples, subsamples were frozen for DNA extraction and 16S rRNA gene amplicon sequencing and stored for FISH analyses. Sampling was carried out with permission from Aalborg Kloak A/S.

### Nucleic acid extraction and 16S rRNA gene amplicon sequencing

Frozen samples (0.5 mL) were used for direct extraction of DNA with the FastDNA® Spin Kit for Soil (MP Biomedicals, Solon, OH, USA), but with a few modifications. The bead beating step was prolonged to 4x40 sec at 6 m/s instead of 40 sec at 6 m/s [11]. Nucleic acids were quantified using the dsDNA BR Assay Kit on a Qubit 2.0 Fluorometer (Invitrogen, Helleup, Denmark), and the purity was checked using NanoDrop ND-1000 Spectrophotometer (NanoDrop Technologies). DNA integrity was verified after gel electrophoresis.

The library preparation protocol was performed as in our previous study on the V1-3 region of the 16S rRNA gene [11]. Library concentrations were measured using the Quant-iT™ PicoGreen® dsDNA Assay Kit (Invitrogen) and quality validated using a TapeStation 2200 using D1K screentapes (Agilent Technologies, Inc., Santa Clara, California, USA). Libraries were pooled in equimolar concentrations and the composite library diluted to 4 nM. The library pool was paired-end (2x250 bp) sequenced on a MiSeq (Illumina, San Diego, CA, USA) using a MiSeq Reagent kit v2 (500 cycle), following the procedure in [13] with the exception of 70% PhiX control library (Illumina, San Diego, CA, USA) spike-in and a final library loading concentration of 20 pM. Custom forward, reverse, and indexing primers were used. The instrument, reagents, and samples were prepared according to the MiSeq User guide rev. D, with the exceptions described in the supplementary material of [13].

### Amplicon bioinformatic processing and analysis

All sequenced libraries were subsampled to 50,000 raw reads and screened for PhiX contamination using bowtie2 v. 2.1.0 [14] with standard settings and all matching reads removed. The potential PhiX contamination is due to the use of an un-indexed PhiX as a quality control, which can result in index carryover from nearby clusters with indexes. Bad quality of the reverse reads prevented merging of the forward and reverse reads, instead the first 225 bp of the forward read from each amplicon was used for further analysis. Reads were quality trimmed using the fastx toolkit (https://github.com/agordon/fastx_toolkit) by removing reads with bases below a Phred score of 20. The reads were dereplicated and formatted for use in the UPARSE workflow [15]. The dereplicated reads were clustered, using the usearch v. 7.0.1090 cluster_otus with default settings. OTU abundance at the approximate species level (97% identity) was estimated using the usearch v. 7.0.1090 usearch_global with -id 0.97. Taxonomy was assigned using the RDP classifier [16] as implemented in the parallel_assign_taxonomy_rdp.py script in QIIME [17], using MiDAS taxonomy version 1.20 [9], which is based on the SILVA taxonomy [18]. The results were analysed in R [19] through the Rstudio IDE (http://www.rstudio.com/) using the ampvis R package (https://github.com/MadsAlbertsen/ampvis).

Principal component analysis (PCA) was conducted using vegan [20] with square root transformed OTU counts, and stability plots were produced using Bray-Curtis dissimilarity. Note that the given OTU abundances are relative read abundances and should not be interpreted directly as *in situ* biomass abundances.

### Fluorescence in situ hybridization (FISH)

The procedures for FISH were performed according to the procedure developed by our colleagues [21] with overnight hybridization. SYTO® 9 green fluorescent nucleic acid stain (Life Technologies^TM^) was used as a universal stain for all Bacteria, and sulfoindocyanine dye (Cy3)-labelled probes were applied to target specific groups. The Dechlo2 [22] and Dech219 probes were optimized for FISH coverage of the genus *Dechloromonas*, essentially as described by our colleagues [23] (see supplementary material as well as S1 and S2 Figs and S1–S4 Tables for details). The probes CFXmix (CFX1223 and GNSB941) [24,25], CFX197 and CFX223 [26] were used to target the phylum Chloroflexi and the Eikelboom morphotype 0092.

### *In situ* staining of bacterial cells with SYTO® 9 green-fluorescent nucleic acid stain

Staining of the bacteria in the biofilm was performed directly on the membrane using SYTO® 9 green-fluorescent nucleic acid stain (Life Technologies^TM^), following the vendor's recommendations. Aliquots of 50 μL of stain were used to cover membrane pieces of 1 cm^2^, which were then incubated for 45 min in the dark before visualization and image acquisition. The thickness of the biofilm was estimated from 20 Z-stacks by marking the top and bottom using confocal laser scanning microscopy (CLSM).

### Microscopy and image acquisition

For FISH, samples were mounted with Citifluor^TM^ antibleaching agent (Citifluor Ltd., London, England). Fluorescent images were recorded using a Zeiss LSM510 CLSM (Carl Zeiss, Jena, Germany) equipped with a Meta-filter and a 63x oil objective. Quantitative FISH (qFISH) was performed by calculating the total biovolume area of cells labelled by the specific probe as a percentage of the area of bacterial cells stained by SYTO® 9, for 20 randomly chosen fields of view, using image analysis software ImageJ 1.45s (Wayne Rasband National Institutes of Health, USA). The average and standard deviations of these values were calculated. The filament index (FI) was assessed according to [27] on a scale from 0 (no filaments) to 5 (excessive amounts).

## Results and Discussion

### MBR operation and biofilm development

**Fig 2:**
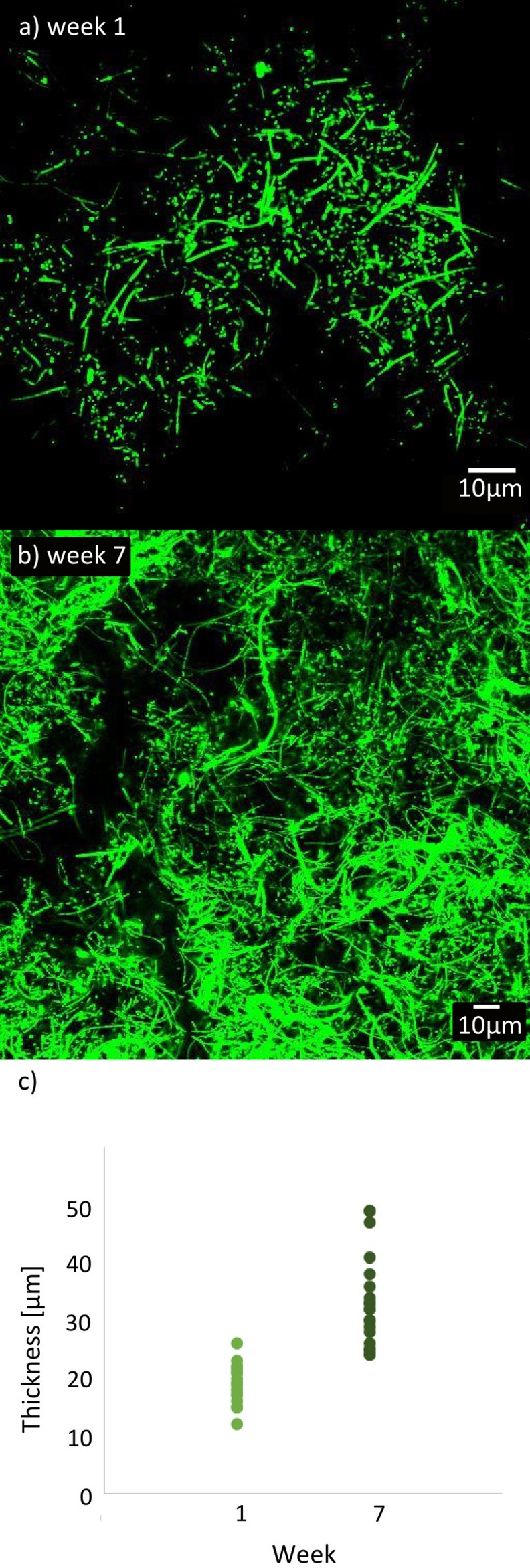
a) SYTO 9 stained cells which shows the amount of filaments at week 1 and b) week 7. The scale bar represents 10 μm. c) The thickness of the biofilm shown as 20 repeated measurements (points) for each of the two weeks.

In this study a membrane bioreactor was run for six months prior to the sampling period to achieve a stable system with regard to permeate flux, nutrient removal, and community structure. The organic loading to the MBR was similar to that of the full-scale plant, and the biomass concentration (MLSS) ranged from 3.6 g/L to 5.4 g/L. The MBR was able to remove 55% of total P and 56% of total N on average (see S5 Table). The daily average permeate flux ranged between 5.3 and 14.4 L/m^2^ h (average 7.8 L/m^2^ h ± 3 L/m^2^ h). Membrane fouling in the system was indicated by the slight decrease in permeate flux at constant TMP (Fig 1B) and a clear build-up of two distinct layers on the mini membrane surface consisting of an outer dark cake layer, that could easily be washed away, and a thin yellow biofilm that adhered strongly to the surface. The thin biofilm was monitored by visualising SYTO® 9 stained cells with a CLSM and showed definite accumulation of cells and microcolonies in a polymer matrix and accompanied by a gradual increase in thickness to approx. 33 μm after 7 weeks (Fig 2). The images 2a) and 2b) also reveal an increasing amount of filamentous bacteria over time. A rapid attachment of bacteria onto a membrane is also shown in other studies [28] and a stratification of the biofilm with microcolonies closest to the membrane and a layer of filaments on top, yielding a porous outer part and a denser bottom layer (close to the membrane), was also observed by Meng and coworkers [29] as well as Gao and coworkers [30], who proposed that the filaments formed a mesh, through which smaller cells and molecules such as nutrients could pass through. The ability of filaments to form porous structures is well described in activated sludge, where they have a significant structural role in the formation of flocs [31].

It was not possible to correlate the development of the biofilm with observed changes in the permeate flux. The dark cake layer was not subjected to further analyses and we cannot exclude its contribution to the decreasing flux, however, the MLSS values for the MBR system (3.6–5.4 g/L) were below the range usually associated with major clogging problems [1].

### Microbial community composition

The bacterial community composition was monitored over time with 16S rRNA gene amplicon sequencing for the MBR bulk sludge, the biofilm, and the CAS sludge. All communities were dominated by the Chloroflexi and Betaproteobacteria, followed by the Actinobacteria, Alphaproteobacteria, Bacteroidetes, Firmicutes, Nitrospirae, Acidobacteria, Gammaproteobacteria and Chlorobi (S3 Fig). S6 Table shows a detailed list of the top 100 species-level OTUs for each sample type, combined yielding 160 OTUs. Overall, the microbial composition of the CAS and MBR were similar and typical for Danish full-scale wastewater treatment systems. Most observed genera belong to the commonly abundant core populations that were revealed by the recent application of molecular methods to large-scale surveys of full-scale activated sludge systems in Denmark [9,32,33]. These surveys revealed a surprisingly stable core community in all EBPR plants, with only 30–40 FISH-defined groups making up 80% of the entire biomass [33] or around 100 abundant genera when identified by 16S rRNA gene amplicon sequencing [9,32]. A similarity between the MBR and CAS systems was also observed with a previous survey of full-scale systems [34]. The presence of few abundant genera across different wastewater treatment systems means that detailed studies of these genera and their influence on plant performance in both CAS and MBR are of general and generic value for full-scale plants, and not only specific for a certain lab-scale reactor set-up. More knowledge about these genera may with time contribute to biofouling control in MBR systems.

### Microbial community dynamics during biofilm maturation

**Fig 3:**
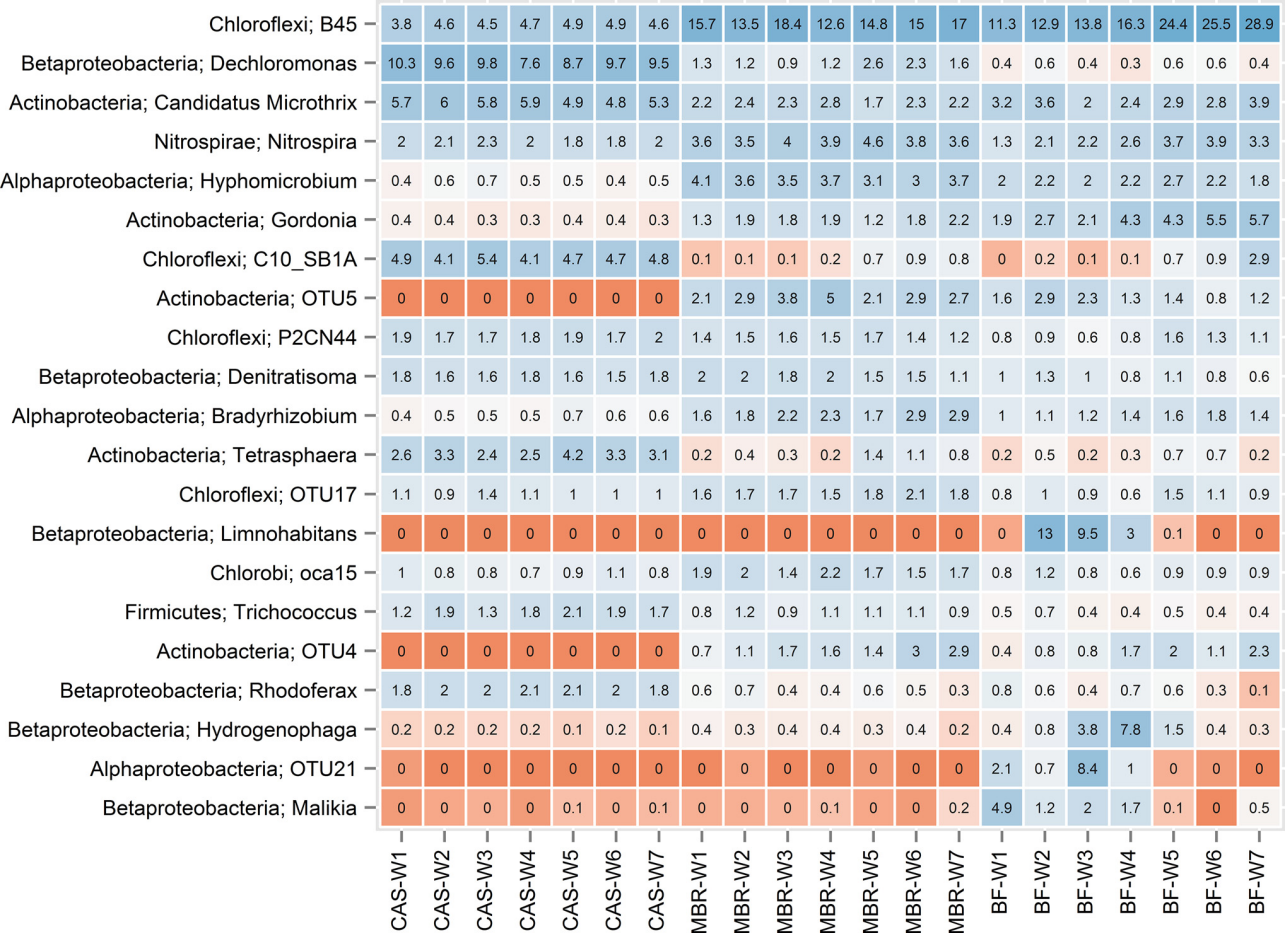
Heatmap showing the microbial composition represented as the 10 most abundant genera of CAS bulk sludge samples (CAS), MBR bulk sludge samples (MBR) and biofilm samples (BF), combined yielding 21 genera. The OTU number is given if it was not classified to the genus level with the MiDAS taxonomy. The data is visualised as a table with underlying colours showing changes and numbers showing the relative read abundances. W1-7 indicate the sampling time (in weeks).

**Fig 4:**
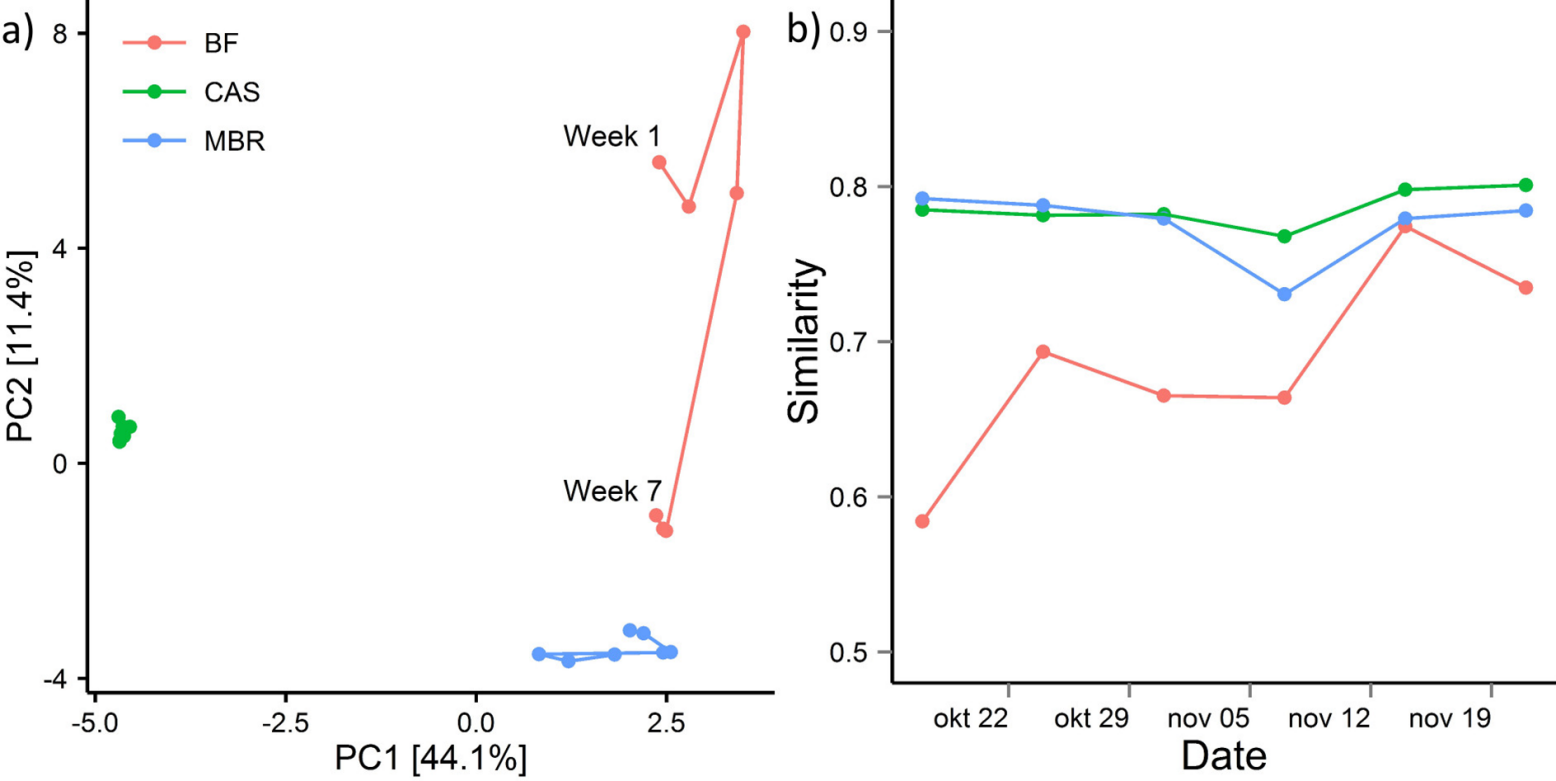
a) PCA plot showing overall differences between CAS bulk sludge samples (CAS, green), MBR bulk sludge samples (MBR, blue) and biofilm samples (BF, red). b) Stability plot showing how similar each sample is to the previous one using Bray-Curtis dissimilarity.

The community dynamics of the biofilm, MBR bulk sludge, and the CAS (feeding into the MBR system) can be seen in Fig 3, which shows the variation over time for the 10 most abundant genera in each sample type; yielding a combined total of 21 genera. In general, the biofilm showed the highest degree of succession, becoming progressively similar to the microbial community in the MBR sludge (Fig 4). This observation is consistent with a previous study, where the communities of the bulk sludge and cake layer were very similar—albeit compared at the phylum level—after long-term operation (188 days) [35]. Interestingly, the biofilm of the current study contained some characteristic OTUs, some of which only occurred in the early biofilm samples, indicating a possible role as pioneer species. These include OTUs within the genera *Limnohabitans*, *Hydrogenophaga*, and *Malikia*, and one OTU (OTU21) within the alphaproteobacterial class (Fig 3). These transient OTUs peaked in abundance at different time points, but disappeared as the biofilm matured, indicating a possible importance for initial adhesion and the establishment of the biofilm. The presence of pioneer species on membrane surfaces is commonly observed, sometimes reportedly colonizing the membrane surface within hours of operation (4 h) [28]. Previous studies have suggested that members of the Betaproteobacteria are important for biofilm formation. Although none of these possible pioneer genera have specifically been reported before in MBR systems, Gao and coworkers [36] also observed members of the Comamonadaceae in the early biofilm. Our recent studies have shown that *Limnohabitans* and other Comamonadaceae are common in incoming wastewater and die off in the activated sludge plant [32]. So instead of being a real pioneer species, their presence may just illustrate their high relative abundance in the incoming wastewater, until a thicker mature biofilm becomes established.

As the biofilm layer matured the betaproteobacterial genera were succeeded by filamentous Chloroflexi and *Gordonia* (see Figs 2 and 4). Members of the Chloroflexi constituted a very high fraction of the biomass in the biofilm (15.2–37.5% relative read abundance) and the MBR sludge (20.8–26%) (Fig 3 and S4 Fig). FISH verified the presence of various Chloroflexi phylotypes, and qFISH analyses were used to confirm the general high abundance of the Chloroflexi phylum (S4 Fig). The most abundant genus in the entire data set was the Chloroflexi B45 phylotype. This genus increased over time in both the biofilm (11.3–28.9%) and the MBR sludge (12.6–18.4%). Using FISH, applying the CFX197 and CFX223 probes, the highly abundant B45 was confirmed to possess the Eikelboom morphotype 0092 [26]. The B45 genus was represented by eight OTUs, of which two were highly abundant (OTU1 and OTU3110), collectively constituting 8.1–20.6% in the biofilm and 10.1–13.5% in the MBR community (S7 Table). Other genera within Chloroflexi were also present, but at considerably lower abundances.

A high abundance of filamentous Chloroflexi in both CAS and MBR communities has commonly been described [33,34,37], where they are often associated with poor settling and reduced filterability, respectively [38,31]. A single study has also described these as abundant in nitrifying biofilms [39]. Information on the dynamics of specific phylotypes of the Chloroflexi has previously been missing due to poor annotation for the phylum in public databases [9], but was highly improved in this study due to use of the curated MiDAS taxonomy. It seems likely that the conditions in the biofilm, i.e. constant aeration and nutrition, perhaps in the form of EPS and SMP, are well suited for some members of the Chloroflexi. Filamentous Chloroflexi have previously been shown to utilize hydrolyzed polysaccharides such as glucose and *N*-actetyl *D*-glucosamine, but also lactose, amino acid mixture, and protein hydrolysate [39,40]. Recent genetic and *in situ* characterisation of the dominant B45 phylotype, now known as “*Candidatus* Promineofilum”, revealed their ability for fermentation with a preference for sugars [41]. The filamentous morphology of the Chloroflexi, protruding from the flocs [38] and biofilm, likely gives them better access to substrates in the bulk liquid. Studies have suggested that filaments have a positive effect on filterability because they are able to degrade parts of the EPS [37,42]. Conversely, several studies link filaments to bulking, bad filterability, and low membrane permeability due to increased resistance [31,29]. As can be seen in S5 Table, the filament index (FI) for the MBR sludge was between 3.0 and 3.5 for all samples, which is approaching FI of 4–5, where membrane resistance is significantly affected [29]. Filaments other than the Chloroflexi, such as *Ca*. Microthrix, were observed at much lower abundances (Fig 3). *Ca*. Microthrix has often been associated with bulking and foaming [31,43], and it may have been involved in the observed foaming of the MBR reactor.

In both the CAS and MBR communities, the *Nitrosomonas* and *Nitrospira* were the abundant known AOBs and NOBs, respectively (see S8 Table). In general, the PAOs were more abundant in the CAS than in MBR, especially *Tetrasphaera* and *Ca*. Accumulibacter (see S9 Table). This is in accordance with the fact that the EBPR process was optimized in the CAS system and not in the MBR reactor. Some members of the *Dechloromonas* behave as PAOs in EBPR systems [44], and the genus has previously been suggested as an important genus for the formation of the biofilm [8]. However, the *Dechloromonas* did not appear to be associated with the biofilm in this study (Fig 3). To confirm their high amplicon read abundance in the CAS system we optimized two FISH probes for the genus *Dechloromonas*, designated Dechlo2 [22] and Dech219 (see Supplementary material for details). Application of these probes to qFISH analyses revealed that the *Dechloromonas* constituted less than 1% of the biovolume in all CAS samples indicating that sequencing-based methods may overestimate their abundance as much as tenfold, which has also been suggested in previous studies [23,45]. There could be several reasons for this, related to, e.g. relative 16S rRNA gene copy number and DNA extraction and amplification biases, highlighting the value of applying amplification and extraction-independent methods for validation of major findings of sequencing based profiles [11,46]. An existing genome for *Dechloromonas aromatica* RCB has four copies of the 16S rRNA gene [47]; suggesting that 16S rRNA gene amplicon sequencing may contribute to an overestimation of their abundance.

The results of this study are preliminary, but demonstrate the potential for high throughput sequencing methods for the monitoring of the communities involved in biofilm formation and fouling. The overall conclusion is that, although putative pioneer species appear, the biofilm becomes very similar to the bulk community with time. Replication of the work with a range of systems is required to investigate the hypothesis of putative biofilm pioneer species in MBR fouling, focusing on their identity and specific potential roles in biofilm formation. Furthermore, this study demonstrates the sequencing resolution and the taxonomic assignment have dramatically improved in our study compared to previous studies of microbial communities in MBR bulk sludge and fouling layer. In addition, our results show that the most abundant genera and OTUs in the MBR were very similar to those commonly observed in full-scale CAS plants, which strongly supports the general notion that most wastewater treatment systems (CAS and MBR) consist of more or less the same abundant OTUs, varying in their ratios between individual plants. This suggests that, to a large extent, these OTUs determine the exact solid-liquid separation properties, including the filtration properties in MBRs. An increased understanding of these MiDAS curated species and their influence on solid-liquid separation properties, their ecology, and how to manipulate their presence, can be used for future optimization of the plants, perhaps also in terms of membrane fouling.

## Supporting Information

S1 Fig Formamide dissociation curves for *Dechloromonas* probes. **a.** Dechlo2; **b.** Dech219. RFU = relative fluorescence units. Values were normalized for each profile such that the fitted curves intersected the y-axis at the same RFU value. Curves were calculated using GraphPad Prism 6 (La Jolla, CA, USA). For target site details see S4 Table.
(PDF)

S2 Fig Phylogenetic tree. Phylogenetic tree of 16S rRNA genes depicting probe coverage of the *Dechloromonas* genus probes. The tree represents the relevant section of the SILVA 16S reference database base tree SSUref_SILVA_111 as visualised in the ARB software. Probe coverage is represented by shading.
(PDF)

S3 Fig Top 10 phyla. Heatmap showing the microbial composition (abundance of taxa) of CAS bulk sludge samples (CAS), MBR bulk sludge samples (MBR) and biofilm samples (BF). The ten most abundant phyla are shown. The data is visualised as a table with underlying colours.
(PDF)

S4 Fig Quantification of Chloroflexi (using 16S rRNA gene amplicon sequencing and qFISH). (PDF)

S1 File *Dechloromonas* probe analysis. (PDF)

S1 Table Specificity, sequences and hybridization conditions for the oligonucleotide probes used. (PDF)

S2 Table *Dechloromonas* probe coverage analysis. (PDF)

S3 Table *Dechloromonas* probe coverage and specificity. (PDF)

S4 Table Mismatch analysis for probes of interest. (PDF)

S5 Table MBR operation and performance data. Operation and performance data for the MBR in the 7-week period when samples were extracted. The average inlet concentrations of total-P and total-N were 4.9 mg/L (range 3.9–5.7 mg/L) and 34.5 mg/L (range 31.0–36.0 mg/L), respectively, during the sampling period.
(PDF)

S6 Table Top 100 OTUs. Abundant OTUs in the three different sample types: CAS bulk sludge samples (CAS), MBR bulk sludge samples (MBR) and biofilm samples (BF). Here shown as the top 100 species-level OTUs (97% sequence similarity cut-off) in each system (combined 160) yielding average cut-off values of 0.16% for CAS, 0.15% for MBR and 0.14% for BF. Abu: Present in two or more samples AND in min 0.1% abundance. Taxonomic assignment according to the MiDAS taxonomy v. 1.20 (p = phylum, o = order, c = class, f = family, g = genus).
(PDF)

S7 Table Chloroflexi in biofilm and MBR. Read abundance of Chloroflexi genera in percentage of all reads in biofilm (BF) and MBR sludge samples. Species-level OTUs are shown. The numbers 1–7 refer to the week of sampling.
(PDF)

S8 Table AOBs and NOBs in CAS and MBR. Read abundance of common AOBs (genus *Nitrosomonas*) and NOBs (genera *Nitrospira* and *Ca*. Nitrotoga) in percentage of all reads in CAS and MBR sludge samples. Species-level OTUs are shown (97% sequence similarity cut-off). The numbers 1–7 refer to the week of sampling.
(PDF)

S9 Table Bacteria involved in P-removal in CAS and MBR. Read abundance of common PAOs (genus *Ca*. Accumulibacter and *Tetrasphaera*) in percentage of all reads in CAS and MBR sludge samples. Species-level OTUs (97% sequence similarity cut-off) are shown. The numbers 1–7 refer to the week of sampling.
(PDF)
