# Supplementary material for: Dynamics of the Fouling Layer Microbial Community in a Membrane Bioreactor
Source: PLoS One. 2016 Jul 11;11(7):e0158811. doi: 10.1371/journal.pone.0158811 (PMC4939938; doi:10.1371/journal.pone.0158811)
Supplement: S1 Fig — a. Dechlo2; b. Dech219. RFU = relative fluorescence units. Values were normalized for each profile such that the fitted curves intersected the y-axis at the same RFU value. Curves were calculated using GraphPad Prism 6 (La Jolla, CA, USA). For target site details see S4 Table. (PDF) [file pone.0158811.s001.pdf]

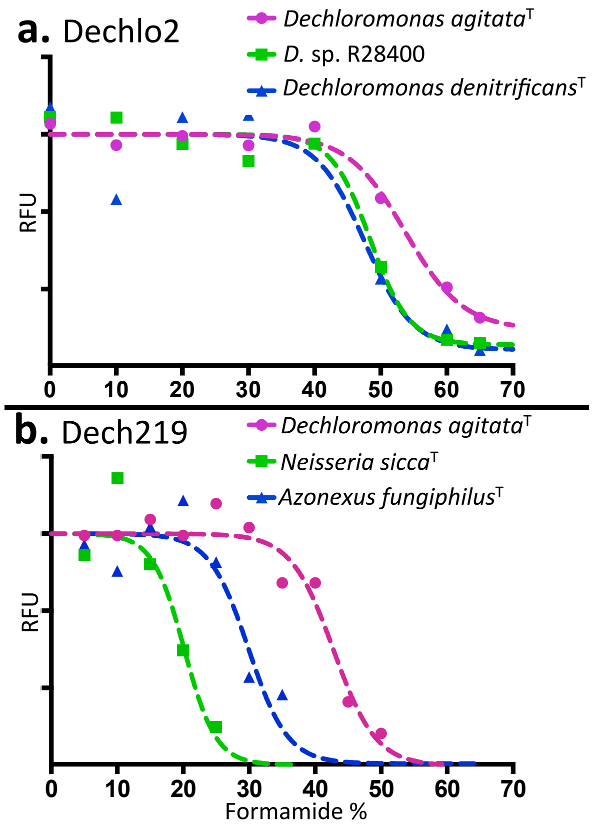

S1 Fig. Formamide dissociation curves for *Dechloromonas* probes. a. Dechlo2; **b.** Dech219. RFU = relative fluorescence units. Values were normalized for each profile such that the fitted curves intersected the y-axis at the same RFU value. Curves were calculated using GraphPad Prism 6 (La Jolla, CA, USA). For target site details see T4 Table.
