# Supplementary material for: Dynamics of the Fouling Layer Microbial Community in a Membrane Bioreactor
Source: PLoS One. 2016 Jul 11;11(7):e0158811. doi: 10.1371/journal.pone.0158811 (PMC4939938; doi:10.1371/journal.pone.0158811)
Supplement: S2 Fig — Phylogenetic tree of 16S rRNA genes depicting probe coverage of the Dechloromonas genus probes. The tree represents the relevant section of the SILVA 16S reference database base tree SSUref_SILVA_111 as visualised in the ARB software. Probe coverage is represented by shading. (PDF) [file pone.0158811.s002.pdf]

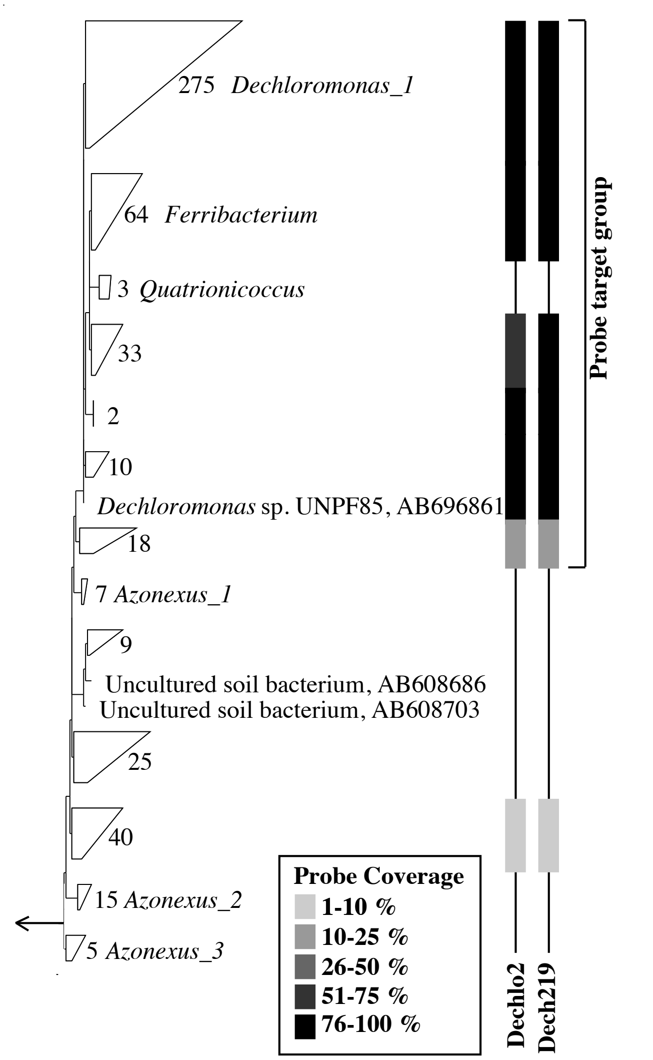

S2 Fig. Phylogenetic tree. Phylogenetic tree of 16S rRNA genes depicting probe coverage of the *Dechloromonas* genus probes. The tree represents the relevant section of the SILVA 16S reference database base tree SSUref_SILVA_111[12] as visualised in the ARB software[1]. Probe coverage is represented by shading.
