# Supplementary material for: Dynamics of the Fouling Layer Microbial Community in a Membrane Bioreactor
Source: PLoS One. 2016 Jul 11;11(7):e0158811. doi: 10.1371/journal.pone.0158811 (PMC4939938; doi:10.1371/journal.pone.0158811)
Supplement: S3 Fig — Heatmap showing the microbial composition (abundance of taxa) of CAS bulk sludge samples (CAS), MBR bulk sludge samples (MBR) and biofilm samples (BF). The ten most abundant phyla are shown. The data is visualised as a table with underlying colours. (PDF) [file pone.0158811.s003.pdf]

	CAS-W1	CAS-W2	CAS-W3	CAS-W4	CAS-W5	CAS-W6	CAS-W7	MBR-W1	MBR-W2	MBR-W3	MBR-W4	MBR-W5	MBR-W6	MBR-W7	BF-W1	BF-W2	BF-W3	BF-W4	BF-W5	BF-W6	BF-W7
Chloroflexi	15.7	14.8	16.7	15.6	15.7	16.4	16.2	23.5	21.7	26	20.8	23.8	23.5	25.6	15.2	18.5	17.5	20.4	31.9	32.7	37.5
Betaproteobacteria	27.3	25.4	25.6	22.9	23	25.3	24.4	14.2	14.9	13	14	14.9	13.4	13.1	19.3	25	23.4	23.2	10.1	6.7	6.1
Actinobacteria	14.9	16.3	14.9	15.3	16.9	15.2	15.5	11.2	14.6	15.9	17.6	14.6	18.1	17	12.8	16.8	12.5	17.6	20.2	22.5	19.7
Alphaproteobacteria	8.2	9.4	9.3	9	9.8	9	9.3	15.1	15	15.1	14.2	14.2	15.6	16	12.8	10.4	20	13.9	13	10.8	8.8
Bacteroidetes	9.7	10.3	10	10.4	9	9.1	8.6	6.7	6.7	5.6	6	7.2	5.7	5	7.4	5.9	4.8	3.9	4.1	3.8	4.5
Firmicutes	4.9	6.1	5.3	6.4	6.8	6.6	6.7	5.3	4.9	3.9	4.4	3.8	4.2	4.2	6.9	4.4	4.3	3.9	4	4.7	5.4
Nitrospirae	2	2.1	2.3	2	1.8	1.8	2	3.6	3.5	4	3.9	4.6	3.8	3.6	1.3	2.1	2.2	2.6	3.7	3.9	3.3
Acidobacteria	1.8	1.5	1.5	1.8	1.9	1.6	1.6	4.6	4.4	3.9	4.5	3.9	3.1	2.9	2	3	1.9	2	2.5	2.3	2.1
Gammaproteobacteria	2.5	2.4	2.1	2.6	2.3	2.1	2.3	2	1.5	1.5	1.6	1.6	1.5	1.3	3.8	2.2	2.9	3.6	1.6	2	1.2
Chlorobi	1.9	1.5	1.6	1.8	1.9	1.8	1.8	2.7	2.7	1.9	3	2.6	2.2	2.5	1.1	1.9	1.3	1.2	1.4	1.6	1.6

S3 Fig. Top 10 phyla. Heatmap showing the microbial composition (abundance of taxa) of CAS bulk sludge samples (CAS), MBR bulk sludge samples (MBR) and biofilm samples (BF). The ten most abundant phyla are shown. The data is visualised as a table with underlying colours.
