# Supplementary material for: Dynamics of the Fouling Layer Microbial Community in a Membrane Bioreactor
Source: PLoS One. 2016 Jul 11;11(7):e0158811. doi: 10.1371/journal.pone.0158811 (PMC4939938; doi:10.1371/journal.pone.0158811)
Supplement: S4 Fig — (PDF) [file pone.0158811.s004.pdf]

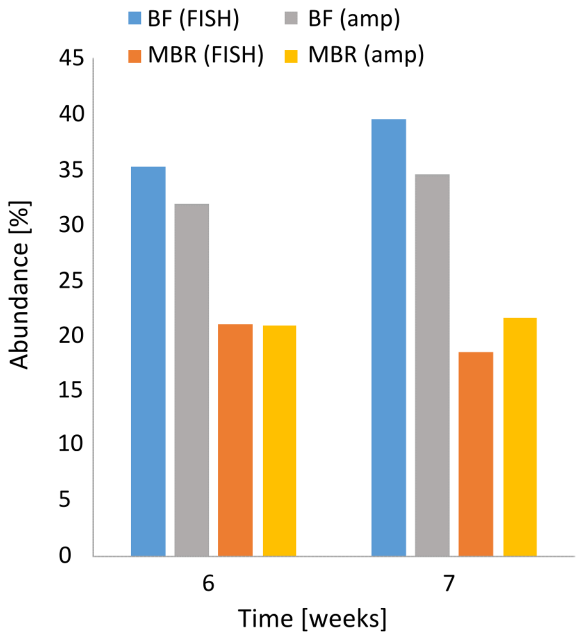

S4 Fig. Quantification of Chloroflexi (using 16S rRNA gene amplicon sequencing and qFISH). Comparison of data from 16S rRNA amplicon sequencing and FISH regarding abundance of Chloroflexi after 6 and 7 weeks. FISH data is shown as % of EUBmix that are positive with the CFXmix probe (CFX1223[16] and GNSB941[17]) and amplicon data (amp) is shown as % of total community reads.
