# Supplementary material for: Dynamics of the Fouling Layer Microbial Community in a Membrane Bioreactor
Source: PLoS One. 2016 Jul 11;11(7):e0158811. doi: 10.1371/journal.pone.0158811 (PMC4939938; doi:10.1371/journal.pone.0158811)
Supplement: S1 File — (PDF) [file pone.0158811.s005.pdf]

S1 file: *Dechloromonas* probe analysis

Probe design and validation

Probes were designed and evaluated with the ARB software package.[1] The mathFISH software[2] was used to calculate hybridization efficiency predictions for perfect and mismatched probe binding. The potential for non-target sequences with indels was also assessed.[3] Competitor probes[4] were designed for single base mismatched non-target sequences. Probe validation and optimization were based on empirically determined formamide dissociation curves[5] where average fluorescent intensities of at least 50 cells calculated with ImageJ software were measured for varied hybridization buffer formamide concentration [FA] from 0-65% (v/v) (Fig. S1). Target and non-target axenic cultures were used for probe optimization where available, otherwise activated sludge was used (see Tab. S1).

Results

As the Dechlo2 probe was originally designed for use in microarrays, it was optimized for FISH and found to have an optimal [FA] of 40% [v/v] (see Fig. S1a). An additional probe, Dech219, was designed by shifting the target site 8 nucleotides and optimized to target the *Dechloromonas* group (Tab. S1 & Fig. S1b). Both the Dechlo2 and Dech219 target a broader phylogenetic cluster that includes the genus *Ferribacterium,* as well as some related unclassified clusters (see Tab. S2 and Fig. S2), with good coverage (Tab. S3). Application of these probes to activated sludge gave fluorescence for rods and coccobacilli, typically present in loose clusters within the flocs. Given the overlap of their target site, the Dechlo2 and Dech219 probe overlap *in situ* could not be directly assessed, but differences were not apparent when visually assessed in several full-scale sludges (data not shown). Although slightly higher coverage is indicated for the Dech219 probe, the Dechlo2 probe has a number of target group weakly mismatched sequences that still give a positive signal, as demonstrated for *Dechloromonas* sp. R-28400 and *Dechloromonas denitrificans*^{T} (see Fig. S1a and Tab. S4). When considering calculated weak terminal mismatched sequences, the Dechlo2 probe gives slightly higher predicted coverage and specificity (data not shown) and is thus recommended for use.

Interestingly, all cells hybridizing the Dechlo2 and Dech219 probes also hybridized with the Nso190 probe, targeting *Nitrosomonas*-*Nitrospira*-related ammonia-oxidizing organisms, despite no evident *in silico* overlap between the two. Investigations were carried out to determine which of the probe sets was mis-hybridizing to non-target organisms. Single base mismatched *Nitrospira*-related sequences were detected with 'Probe Match' (RDP II)[6] (Tab. S4). However, these sequences were all partial (<1200 bp), from soil, and almost entirely generated by a single study. The single base mismatch is the same for both Dechlo2 and Dech219 probes (*E. coli* position 223). In addition, application of unlabelled competitor probes specific to this mismatch made no evident difference to binding of the *Dechloromonas* probes in the activated sludge samples tested. Few *Dechloromonas*-related sequences have a single base mismatch with the Nso190 probe, although almost all have 2 terminal mismatches (Tab. S4). Application of an unlabelled competitor probe specific for these mismatches always eliminated *in situ* crossover between the Nso190 and *Dechloromonas* probes. This was confirmed by applying the probe to *D. agitata*, which gave bright positive fluorescence at 40% [FA] and slightly higher than background levels at 55%. These analyses indicate that it is the Nso190 probe that is mis-hybridizing with *Dechloromonas*-related organisms giving the overlap in *in situ* coverage. Since the initial description of the Nso190 probe suggested its application at 55% [FA], several studies have suggested lower concentrations, ranging from 20-50% [FA], be used due to low and inconsistent

signal.[7,8,9] Since signal was detected when the Nso190 probe was applied to *D. agitata* all the way up to 55% [FA], it is recommended that the competitor probe designed here be used, regardless of the [FA] selected, to prevent the probe from mishybridizing to *Dechloromonas*-related organisms.

References:

1. Ludwig W., Strunk O., Westram R., Richter L., Meier H. Yadhukumar, *et al.* ARB: a software environment for sequence data. *Nucleic Acids Res* 2004; 32; 1363-1371.

2. Yilmaz L.S., Parnerkar S., Noguera D.R. mathFISH, a web tool that uses thermodynamics-based mathematical models for in silico evaluation of oligonucleotide probes for fluorescence in situ hybridization. *Appl Environ Microbiol* 2011; 77; 1118-22.

3. McIlroy S.J., Tillett D., Petrovski S., Seviour R.J. Non-target sites with single nucleotide insertions or deletions are frequently found in 16S rRNA sequences and can lead to false positives in fluorescence*in situ* hybridization (FISH). *Environ Microbiol* 2011; 13; 38-47.

4. Manz W., Amann R., Ludwig W., Wagner M., Scheifer K. Phylogenetic oligodeoxynucleotide probes for the major subclasses of proteobacteria: problems and solutions. *Systematic and Applied Microbiology* 1992; 15; 593-600.

5. Daims H., Stoecker K., Wagner M. Fluorescence *in situ* hybridization for the detection of prokaryotes. In: *Molecular Microbial Ecology.* Taylor & Francis: New York; 2005; pp 213-239.

6. Cole J.R., Wang Q., Cardenas E., Fish J., Chai B., Farris R.J*., et al.* The Ribosomal Database Project: improved alignments and new tools for rRNA analysis. *Nucleic Acids Res* 2009; 37; D141-5.

7. Konuma S.; Satoh H.; Mino T.; Matsuo T. Comparison of enumeration methods for ammonia-oxidizing bacteria. *Water Sci Technol* 2001; 43; 107-114.

8. Pynaert K.; Smets B.F.; Wyffels S.; Beheydt D.; Siciliano S.D.; Verstraete W. Characterization of an autotrophic nitrogen-removing biofilm from a highly loaded lab-scale rotating biological contactor. *Appl Environ Microbiol* 2003; 69, 3626-3635.

9. Persson F.; Wik T.; Sorensson F.; Hermansso M. Distribution and activity of ammonia oxidizing bacteria in a large full-scale trickling filter. *Water Res* 2002; 36, 1439-1448.

10. Sanguin H.; Herrera A.; Oger-Desfeux C.; Dechesne A.; Simonet P.; Navarro E.; *et al.* Development and validation of a prototype 16S rRNA-based taxonomic microarray for Alphaproteobacteria. *Environ Microbiol* 2006; 8, 289–307.

11. Mobarry B.; Wagner M.; V U; Rittmann B.; Stahl D. Phylogenetic probes for analyzing abundance and spatial organization of nitrifying bacteria. *Appl Environ Microbiol* 1996; 62, 2156-2162.

12. Pruesse E.; Quast C.; Knittel K.; Fuchs B.; Ludwig W.; Peplies J.*; et al.* SILVA: a comprehensive online resource for quality checked and aligned ribosomal RNA sequence data compatible with ARB. *Nucleic Acids Res* 2007; 35, 188-196.

13. DeSantis T.Z.; Hugenholtz P.; Larsen N.; Rojas M.; Brodie E.L.; Keller K.*; et al.* Greengenes, a chimera-checked 16S rRNA gene database and workbench compatible with ARB. *Appl Environ Microbiol* 2006; 72; 5069-5072.

14. Eikelboom D.H. *Process control of activated sludge plants by microscopic investigation*. ASIS; 2000

15. McIlroy S.J.; Saunders A.M.; Albertsen, M.; Nierychlo, M.; McIlroy, B.; Hansen, A.A.; Karst, S.M.; Nielsen, J.L.; Nielsen, P.H. MiDAS: the field guide to the microbes of activated sludge. *Database* 2015; 2015: bav062.

16. Bjornsson L.; Hugenholtz P., Tyson, G.W.; Blackall, L.L. Filamentous *Chloroflexi* (greennon-sulfur bacteria) are abundant in wastewater treatment processes with biological nutrient removal. *Microbiology* 2002; 148; 2309-2318.

17. Gich, F.; Garcia-Gil, J.; Overmann, J. Previously unknown and phylogenetically diverse members of the green nonsulfur bacteria are indigenous to freshwater lakes. *Arch Microbiol* 2001; 117; 1–10.
