# Supplementary material for: Dynamics of the Fouling Layer Microbial Community in a Membrane Bioreactor
Source: PLoS One. 2016 Jul 11;11(7):e0158811. doi: 10.1371/journal.pone.0158811 (PMC4939938; doi:10.1371/journal.pone.0158811)
Supplement: S1 Table — (PDF) [file pone.0158811.s006.pdf]

S1 Table: Specificity, sequences and hybridization conditions for the oligonucleotide probes used.

Probe Name	*E.coli* pos.	Target group	Sequence (5' -3')	Validated against	% FA*	Reference
**Dechlo2**	**211-230**	***Dechloromonas-Ferribacterium*-related**	**GCT CAA TCA GCG CAA GGT CT**	***Dechloromonas agitata* str. CKB^{T}**<br>***Dechloromonas* sp. R284000**<br>***Dechloromonas denitrificans* str. ED1^{T}**	**40**	**10**
Dechlo2_c1	211-230	Competitor probe for Dechlo2	GCT CAA TGA GCG CAA GGT CT	-	-	This study
**Dech219 ****	**219-236**	***Dechloromonas-Ferribacterium*-related**	**TCG GCC GCT CAA TCA GCG**	***Dechloromonas agitata* str. CKB^{T}**	**40**	**This study**
Dech219_c1	219-236	Competitor probe for Dech219	TCG GCC GCT CAA ACA GCG	*Azonexus fungiphilus* str. BS5-8^{T}	-	This study
Dech219_c2	219-236	Competitor probe for Dech219	TCG GCC GCT CYA TCA GCG	*Neisseria sicca* str. DSM17713^{T}	-	This study
Dech219_c3	219-236	Competitor probe for Dech219	TCG GCC GCT CAA TGA GCG	-	-	This study
**Nso190**	**190-208**	***Nitrosomonas*-related**	**CGA TCC CCT GCT TTT CTC C**	**-**	**55**	**11**
Nso190_c1	190-208	Competitor probe for Nso190	CGA TCC CCT GCT TTC CTG C	***Dechloromonas agitata* str. CKB^{T}**<br>Aalborg East WWTP	-	This study

* Recommended formamide concentration in the hybridization buffer. **Dech219_c1 and Dech219_c2 were shown not to be required to prevent detectable binding of the Dech219 probe to the respective sequence they target at the recommended FA of 40% (see Fig S1b). Therefore, Dech219 should be applied only with competitor Dech219_c3 as the mismatch has not been empirically shown to prevent probe binding.
