# Supplementary material for: Dynamics of the Fouling Layer Microbial Community in a Membrane Bioreactor
Source: PLoS One. 2016 Jul 11;11(7):e0158811. doi: 10.1371/journal.pone.0158811 (PMC4939938; doi:10.1371/journal.pone.0158811)
Supplement: S2 Table — (PDF) [file pone.0158811.s007.pdf]

S2 Table: *Dechloromonas* probe coverage analysis

	RDP		SILVA	
**Phylogenetic group**	**Dechlo2**	**Dech219**	**Dechlo2**	**Dech219**
Dechloromonas	227/319 (71%)	244/319 (76%)	209/275 (76%)	221/275 (80%)
Ferribacterium	47/49 (96%)	49/49 (100%)	62/64 (97%)	63/64 (98%)
Zoogloea	1/552 (<1%)	1/552 (<1%)	2/242 (<1%)	2/242 (<1%)
Uliginosidbacterium	-	-	1/26 (4%)	1/26 (4%)
Azospira	-	-	-	1/219 (<1%)
Unclassified *Rhodocyclaceae*	28	33	45	52
**Total *Rhodocyclaceae***	**303**	**327**	**318**	**338**
Massilia	2/1706 (<1%)	2/1706 (<1%)	-	-
Neisseria	-	1/8464 (<1%)	-	-
Limnohabitans	-	-	2/711 (<1%)	2/711 (<1%)
Sphaerotilus	-	-	1/169 (<1%)	1/169 (<1%)
Janthinobacterium	-	-	2/1976 (<1%)	2/1976 (<1%)
Undibacterium	-	-	1/242 (<1%)	1/242 (<1%)
**Total non-*Rhodocyclaceae***	**6**	**9**	**12**	**12**

Values as fractions of group hits/total number classified in-group, % are given in parentheses. RDP = Ribosomal Database – Release 10, update 32 (analysis includes 'good' sequences; >1200bp)[6]; SILVA = SSU release 111 (analysis excludes highlighted suspect sequences ('color group 1'))[12]; Greengenes = release October 2012[13].
