# Supplementary material for: Dynamics of the Fouling Layer Microbial Community in a Membrane Bioreactor
Source: PLoS One. 2016 Jul 11;11(7):e0158811. doi: 10.1371/journal.pone.0158811 (PMC4939938; doi:10.1371/journal.pone.0158811)
Supplement: S3 Table — (PDF) [file pone.0158811.s008.pdf]

S3 Table: *Dechloromonas* probe coverage and specificity

Probe	Group coverage*		Non-target hits	
			Total	AS assoc.**
Dechlo2	308/406	76%	21	10
Dech219	326/406	80%	24	13

Analysis with the SILVA = SSU release 111 (SSUref_SILVA_111: excludes highlighted suspect sequences ('color group 1'))[12]. *Group as defined in Fig. S2 to include *Dechloromonas, Ferribacterium* and several related unclassified phylogenetic clusters. ** Sequences from activated sludge-related environments.
