# Supplementary material for: Dynamics of the Fouling Layer Microbial Community in a Membrane Bioreactor
Source: PLoS One. 2016 Jul 11;11(7):e0158811. doi: 10.1371/journal.pone.0158811 (PMC4939938; doi:10.1371/journal.pone.0158811)
Supplement: S4 Table — (PDF) [file pone.0158811.s009.pdf]

S4 Table: Mismatch analysis for probes of interest.

<table>
<tr><th>Probe</th><th>Binding site (acc. no.)</th><th>Probe alignment*</th><th>[FA]m %*</th></tr>
<tr><td>Dechlo2</td><td>D. agitata (NR_024884)</td><td>...............................
PROBE--3'TCTGGAACGCGACTAACTCG5'
.........||||||||||||||||||||
TARGET-5'AGACCTTGCGCTGATTGAGC3'
...............................</td><td>32.6</td></tr>
<tr><td></td><td>D. sp. R-28400 (AM084133)</td><td>.........T.....................
PROBE--3'.CTGGAACGCGACTAACTCG5'
..........|||||||||||||||||||
TARGET-5'.GACCTTGCGCTGATTGAGC3'
.........G.....................</td><td>29.3</td></tr>
<tr><td></td><td>D. denitrificans (NR_042090)</td><td>.........T.T...................
PROBE--3'.C.GGAACGCGACTAACTCG5'
..........|.|||||||||||||||||
TARGET-5'.G.CCTTGCGCTGATTGAGC3'
.........G.G...................</td><td>5.3</td></tr>
<tr><td></td><td>Nitrospira-related clone (AY631528)</td><td>.....................C.........
PROBE--3'TCTGGAACGCGA.TAACTCG5'
.........||||||||||||.|||||||
TARGET-5'AGACCTTGCGCT.ATTGAGC3'
.....................C.........</td><td>16.5</td></tr>
<tr><td>Dech219</td><td>D. agitata (NR_024884)</td><td>.............................
PROBE--3'GCGACTAACTCGCCGGCT5'
.........||||||||||||||||||
TARGET-5'CGCTGATTGAGCGGCCGA3'
.............................</td><td>33.4</td></tr>
<tr><td></td><td>Neisseria sicca (AJ247248)</td><td>..............T..............
PROBE--3'GCGAC.AACTCGCCGGCT5'
.........|||||.||||||||||||
TARGET-5'CGCTG.TTGAGCGGCCGA3'
..............T..............</td><td>18.9</td></tr>
</table>

<table>
<tr><th></th><th></th><th></th><th></th></tr>
<tr><td></td><td>Azonexus fungiphilus (AF011350)</td><td>................A..........
PROBE--3'GCGACTA.CTCGCCGGCT5'
.........|||||||.||||||||||
TARGET-5'CGCTGAT.GAGCGGCCGA3'
................G..........</td><td>-</td></tr>
<tr><td></td><td>Nitrospira-related clone (AY631528)</td><td>.............C.............
PROBE--3'GCGA.TAACTCGCCGGCT5'
.........||||.|||||||||||||
TARGET-5'CGCT.ATTGAGCGGCCGA3'
.............C.............</td><td>1.7</td></tr>
<tr><td>Nso190</td><td>Nitrosomonas europaea (NR_074774)</td><td>............................
PROBE--3'CCTCTTTTCGTCCCCTAGC5'
.........|||||||||||||||||||
TARGET-5'GGAGAAAAGCAGGGGATCG3'
............................</td><td>46.1</td></tr>
<tr><td></td><td>D. agitata (NR_024884)</td><td>..........C..T..............
PROBE--3'C.TC.TTTCGTCCCCTAGC5'
.........|.||.||||||||||||||
TARGET-5'G.AG.AAAGCAGGGGATCG3'
..........C..G..............</td><td>19.3</td></tr>
</table>

* Predicted with MathFISH.[2] [FA]m = predicted formamide melting point.
