# Supplementary material for: Dynamics of the Fouling Layer Microbial Community in a Membrane Bioreactor
Source: PLoS One. 2016 Jul 11;11(7):e0158811. doi: 10.1371/journal.pone.0158811 (PMC4939938; doi:10.1371/journal.pone.0158811)
Supplement: S5 Table — Operation and performance data for the MBR in the 7-week period when samples were extracted. The average inlet concentrations of total-P and total-N were 4.9 mg/L (range 3.9–5.7 mg/L) and 34.5 mg/L (range 31.0–36.0 mg/L), respectively, during the sampling period. (PDF) [file pone.0158811.s010.pdf]

S5 Table: MBR operation and performance data. Operation and performance data for the MBR in the 7-week period when samples were extracted. The average inlet concentrations of total-P and total-N were 4.9 mg/L (range 3.9-5.7 mg/L) and 34.5 mg/L (range 31.0-36.0 mg/L), respectively, during the sampling period.

			**Online MBR measurements (daily averages)**			**Outlet MBR**			**Microscopy**
Date	Week	SS [g/L]	Permeate flux [L/h]	Temperature [C]	O_2 [mg/L]	NH_4 [mg N/L]	NO_3 [mg N/L]	PO_4 [mg P/L]	Filament Index*
2012-10-10	1	3.6	321	18.0	2.2	0.07	10	2.3	-
2012-10-17	2	3.7	269	18.3	1.9	-	-	-	-
2012-10-24	3	3.8	245	19.0	1.7	0.04	7.5	2.1	3.5
2012-10-31	4	4.7	213	16.4	0.8	0.15	7.5	2.1	3.5
2012-11-07	5	4.5	275	16.9	3.7	0.02	22.8	2.1	3.0
2012-11-14	6	5.0	280	17.1	3.2	0.005	23.3	2.1	3.5
2012-11-21	7	5.4	577	15.9	1.4	2.9	17.9	2.7	3.5

– : data not available, *Assigned according to Eikelboom (2000)[14]
