# Supplementary material for: Dynamics of the Fouling Layer Microbial Community in a Membrane Bioreactor
Source: PLoS One. 2016 Jul 11;11(7):e0158811. doi: 10.1371/journal.pone.0158811 (PMC4939938; doi:10.1371/journal.pone.0158811)
Supplement: S6 Table — Abundant OTUs in the three different sample types: CAS bulk sludge samples (CAS), MBR bulk sludge samples (MBR) and biofilm samples (BF). Here shown as the top 100 species-level OTUs (97% sequence similarity cut-off) in each system (combined 160) yielding average cut-off values of 0.16% for CAS, 0.15% for MBR and 0.14% for BF. Abu: Present in two or more samples AND in min 0.1% abundance. Taxonomic assignment according to the MiDAS taxonomy v. 1.20 (p = phylum, o = order, c = class, f = family, g = genus). (PDF) [file pone.0158811.s011.pdf]

S6 Table: Top 100 OTUs. Abundant OTUs in the three different sample types: CAS bulk sludge samples (CAS), MBR bulk sludge samples (MBR) and biofilm samples (BF). Here shown as the top 100 species-level OTUs (97 % sequence similarity cut-off) in each system (combined 160) yielding average cut-off values of 0.16% for CAS, 0.15% for MBR and 0.14% for BF. Abu: Present in two or more samples AND in min 0.1% abundance. Taxonomic assignment according to the MiDAS taxonomy v. 1.20 (p = phylum, o = order, c = class, f = family, g = genus).[15]

				**CAS**				**MBR**				**BF**			
	**OTU**	**Taxonomy**		**Abu**	**Min**	**Max**	**Avg**	**Abu**	**Min**	**Max**	**Avg**	**Abu**	**Min**	**Max**	**Avg**
1	OTU_32	p__Acidobacteria	f__DS-100_OTU_32	X	0.10	0.13	0.11	X	0.46	0.92	0.66	X	0.26	0.49	0.35
2	OTU_12	p__Acidobacteria	o__Subgroup 6_OTU_12	X	0.15	0.19	0.17	X	1.19	2.02	1.63	X	0.60	1.15	0.77
3	OTU_177	p__Acidobacteria	g__SBRFL126	X	0.06	0.13	0.09	X	0.17	0.26	0.20	X	0.06	0.18	0.13
4	OTU_123	p__Acidobacteria	g__S-Btb7_22	X	0.20	0.31	0.24	X	0.06	0.15	0.10		0.03	0.06	0.05
5	OTU_178	p__Actinobacteria	g__Aeromicrobium	X	0.04	0.37	0.17		0.00	0.02	0.01		0.00	0.02	0.01
6	OTU_26	p__Actinobacteria	g__B1-K2-141		0.03	0.07	0.05	X	0.10	0.19	0.15	X	0.57	2.19	1.11
7	OTU_6	p__Actinobacteria	g__Candidatus Microthrix	X	3.81	4.26	4.05	X	0.12	0.70	0.37	X	0.09	1.74	0.54
8	OTU_13	p__Actinobacteria	g__Candidatus Microthrix	X	1.00	1.68	1.30	X	1.24	2.50	1.90	X	1.99	3.31	2.43
9	OTU_70	p__Actinobacteria	f__Nocardiaceae_OTU_70		0.01	0.04	0.03	X	0.06	0.12	0.09	X	0.07	0.33	0.20
10	OTU_5	p__Actinobacteria	f__YNPFFP40_OTU_5		0.00	0.00	0.00	X	1.75	4.78	2.96	X	1.02	3.02	1.74
11	OTU_30	p__Actinobacteria	g__Fodinibacter	X	0.42	0.70	0.52	X	0.08	0.22	0.14	X	0.05	0.39	0.19
12	OTU_45	p__Actinobacteria	g__Fodinicola	X	0.36	0.57	0.45	X	0.01	0.18	0.07	X	0.04	1.74	0.40
13	OTU_3	p__Actinobacteria	g__Gordonia	X	0.25	0.36	0.30	X	1.24	2.26	1.73	X	1.95	5.36	3.57
14	OTU_16	p__Actinobacteria	g__HTG5		0.03	0.08	0.06	X	0.66	1.32	0.88	X	0.34	1.22	0.76
15	OTU_50	p__Actinobacteria	g__HTG5	X	0.14	0.29	0.21	X	0.11	0.30	0.19	X	0.08	0.25	0.14
16	OTU_56	p__Actinobacteria	g__HTG5	X	0.14	0.26	0.20	X	0.14	0.31	0.26	X	0.11	0.31	0.19
17	OTU_103	p__Actinobacteria	g__K2-78		0.01	0.03	0.02	X	0.13	0.21	0.17	X	0.14	0.17	0.16
18	OTU_69	p__Actinobacteria	g__Leucobacter	X	0.39	0.51	0.45	X	0.06	0.16	0.10		0.02	0.07	0.05
19	OTU_27	p__Actinobacteria	g__ML817J-10		0.00	0.02	0.01	X	0.52	0.76	0.61	X	0.26	1.09	0.54
20	OTU_87	p__Actinobacteria	g__Mycobacterium	X	0.11	0.18	0.15		0.04	0.08	0.06	X	0.07	0.29	0.18
21	OTU_54	p__Actinobacteria	g__Nocardioides	X	0.10	0.29	0.21	X	0.13	0.43	0.30	X	0.08	0.31	0.20
22	OTU_4	p__Actinobacteria	o__Acidimicrobiales_OTU_4		0.00	0.01	0.00	X	0.79	2.86	1.77	X	0.38	2.36	1.35

23	OTU_79	p__Actinobacteria	o__Corynebacteriales_OTU_79	X	0.16	0.26	0.22	X	0.06	0.11	0.08	X	0.10	0.23	0.17
24	OTU_57	p__Actinobacteria	g__Tessaracoccus	X	0.08	0.19	0.13	X	0.09	0.22	0.15	X	0.12	0.30	0.19
25	OTU_24	p__Actinobacteria	g__Tetrasphaera	X	2.32	3.56	2.71	X	0.18	1.17	0.55	X	0.13	0.54	0.29
26	OTU_163	p__Actinobacteria	g__Tetrasphaera	X	0.13	0.24	0.16		0.00	0.07	0.02		0.00	0.04	0.02
27	OTU_102	p__Bacteroidetes	c__Bacteroidetes_OTU_102	X	0.19	0.32	0.25		0.02	0.15	0.07		0.01	0.08	0.03
28	OTU_25	p__Bacteroidetes	g__Candidatus Epiflobacter	X	0.68	0.89	0.78	X	0.18	0.45	0.32	X	0.13	0.43	0.23
29	OTU_66	p__Bacteroidetes	g__CYCU-0281	X	0.29	0.45	0.37	X	0.01	0.25	0.10	X	0.03	0.19	0.08
30	OTU_91	p__Bacteroidetes	g__CYCU-0281	X	0.17	0.33	0.25		0.02	0.13	0.05		0.01	0.04	0.02
31	OTU_99	p__Bacteroidetes	g__CYCU-0281	X	0.13	0.31	0.21	X	0.10	0.25	0.15	X	0.10	0.19	0.14
32	OTU_106	p__Bacteroidetes	f__Cytophagaceae_OTU_106		0.02	0.05	0.03	X	0.10	0.26	0.19	X	0.05	0.19	0.10
33	OTU_196	p__Bacteroidetes	f__Saprospiraceae_OTU_196	X	0.16	0.20	0.19		0.00	0.02	0.01		0.00	0.01	0.00
34	OTU_46	p__Bacteroidetes	g__Ferruginibacter	X	0.32	0.61	0.45	X	0.09	0.34	0.18	X	0.04	0.20	0.12
35	OTU_111	p__Bacteroidetes	g__MK04	X	0.36	0.45	0.41		0.00	0.05	0.02		0.00	0.01	0.01
36	OTU_114	p__Bacteroidetes	g__PHOS-HE28		0.01	0.02	0.02	X	0.15	0.27	0.18	X	0.08	0.16	0.13
37	OTU_115	p__Bacteroidetes	g__PHOS-HE28	X	0.16	0.27	0.21		0.01	0.10	0.05		0.01	0.04	0.02
38	OTU_124	p__Bacteroidetes	g__PHOS-HE28	X	0.05	0.09	0.07	X	0.09	0.24	0.14	X	0.07	0.23	0.14
39	OTU_127	p__Bacteroidetes	g__PHOS-HE28	X	0.15	0.24	0.20		0.01	0.06	0.03		0.00	0.02	0.01
40	OTU_128	p__Bacteroidetes	g__PHOS-HE31	X	0.24	0.33	0.27	X	0.06	0.16	0.10	X	0.04	0.11	0.07
41	OTU_52	p__Bacteroidetes	g__QEDR3BF09	X	0.20	0.32	0.25	X	0.07	0.50	0.22	X	0.07	0.28	0.17
42	OTU_169	p__Bacteroidetes	g__QEDR3BF09	X	0.20	0.31	0.23		0.01	0.04	0.02		0.00	0.02	0.01
43	OTU_257	p__Candidate division TM7	c__Candidate division TM7_OTU_257	X	0.10	0.23	0.17		0.00	0.01	0.00		0.00	0.01	0.00
44	OTU_116	p__Chlorobi	f__OPB56_OTU_116	X	0.14	0.18	0.16		0.01	0.09	0.04		0.01	0.03	0.02
45	OTU_28	p__Chlorobi	g__oca15	X	0.28	0.39	0.32	X	0.50	0.86	0.69	X	0.27	0.54	0.36
46	OTU_36	p__Chlorobi	g__oca15	X	0.32	0.44	0.36	X	0.65	0.93	0.76	X	0.29	0.55	0.41
47	OTU_306	p__Chlorobi	g__oca15	X	0.09	0.13	0.10	X	0.11	0.22	0.17	X	0.06	0.14	0.09
48	OTU_1	p__Chloroflexi	g__B45	X	2.11	2.78	2.50	X	6.24	10.17	8.31	X	5.19	10.66	7.24
49	OTU_15	p__Chloroflexi	g__B45	X	0.40	0.56	0.48	X	1.41	1.61	1.50	X	1.16	2.12	1.62
50	OTU_39	p__Chloroflexi	g__B45		0.02	0.04	0.03	X	0.16	0.29	0.22	X	0.24	1.19	0.72

51	OTU_55	p__Chloroflexi	g__B45		0.05	0.08	0.07	X	0.23	0.42	0.32	X	0.30	1.17	0.69
52	OTU_117	p__Chloroflexi	g__B45	X	0.18	0.26	0.23	X	0.53	0.77	0.64	X	0.38	0.81	0.60
53	OTU_1498	p__Chloroflexi	g__B45	X	0.10	0.15	0.13	X	0.33	0.58	0.43	X	0.33	0.69	0.48
54	OTU_3110	p__Chloroflexi	g__B45	X	0.68	0.82	0.73	X	2.66	3.44	2.93	X	2.92	9.92	5.54
55	OTU_5102	p__Chloroflexi	g__B45	X	0.29	0.42	0.36	X	0.62	1.01	0.83	X	1.04	3.05	1.88
56	OTU_14	p__Chloroflexi	g__C10_SB1A	X	3.10	3.80	3.47	X	0.04	0.58	0.26	X	0.03	2.12	0.49
57	OTU_1679	p__Chloroflexi	g__C10_SB1A	X	0.98	1.46	1.20	X	0.04	0.22	0.12	X	0.04	0.45	0.18
58	OTU_82	p__Chloroflexi	g__Candidatus Sarcinathrix	X	0.26	0.35	0.29		0.04	0.11	0.08		0.03	0.10	0.06
59	OTU_108	p__Chloroflexi	f__Caldilineaceae_OTU_108		0.01	0.04	0.03	X	0.12	0.22	0.16	X	0.08	0.13	0.10
60	OTU_61	p__Chloroflexi	f__Caldilineaceae_OTU_61	X	0.09	0.12	0.10	X	0.29	0.36	0.32	X	0.16	0.27	0.20
61	OTU_96	p__Chloroflexi	f__Caldilineaceae_OTU_96		0.04	0.06	0.05	X	0.08	0.18	0.13	X	0.15	0.25	0.19
62	OTU_62	p__Chloroflexi	g__mle1-48		0.04	0.08	0.06	X	0.23	0.38	0.31	X	0.10	0.28	0.17
63	OTU_17	p__Chloroflexi	o__AKYG1722_OTU_17	X	0.90	1.17	1.05	X	1.49	1.96	1.68	X	0.65	1.42	0.96
64	OTU_29	p__Chloroflexi	g__P2CN44	X	0.42	0.56	0.50	X	0.39	0.56	0.49	X	0.22	0.46	0.33
65	OTU_53	p__Chloroflexi	g__P2CN44	X	0.32	0.45	0.37	X	0.16	0.23	0.20	X	0.06	0.22	0.14
66	OTU_74	p__Chloroflexi	g__P2CN44	X	0.19	0.24	0.22	X	0.18	0.28	0.23	X	0.07	0.17	0.10
67	OTU_59	p__Chloroflexi	g__SBR1029	X	0.40	0.51	0.44	X	0.07	0.22	0.13	X	0.02	0.11	0.06
68	OTU_3702	p__Chloroflexi	g__SBR1029		0.03	0.05	0.04	X	0.13	0.29	0.19		0.05	0.11	0.08
69	OTU_140	p__Chloroflexi	g__WCHB1-50	X	0.12	0.26	0.19	X	0.16	0.29	0.21	X	0.08	0.22	0.13
70	OTU_153	p__Firmicutes	g__Clostridium sensu stricto 1	X	0.12	0.19	0.15	X	0.12	0.20	0.15	X	0.17	0.28	0.23
71	OTU_203	p__Firmicutes	g__Clostridium sensu stricto 1	X	0.11	0.19	0.15	X	0.14	0.19	0.16	X	0.24	0.42	0.35
72	OTU_63	p__Firmicutes	g__p-55-a5	X	0.31	0.46	0.40	X	0.23	0.39	0.30	X	0.48	1.03	0.69
73	OTU_84	p__Firmicutes	g__p-55-a5	X	0.12	0.18	0.15	X	0.15	0.19	0.16	X	0.18	0.46	0.30
74	OTU_237	p__Firmicutes	g__p-55-a5	X	0.19	0.25	0.21	X	0.12	0.18	0.15	X	0.22	0.49	0.34
75	OTU_89	p__Firmicutes	g__Subdoligranulum	X	0.21	0.28	0.25	X	0.12	0.25	0.19	X	0.11	0.23	0.17
76	OTU_20	p__Firmicutes	g__Trichococcus	X	1.17	1.93	1.65	X	0.89	1.19	0.99	X	0.41	0.66	0.50
77	OTU_93	p__Firmicutes	g__Turicibacter	X	0.07	0.11	0.09	X	0.10	0.16	0.13	X	0.14	0.41	0.21
78	OTU_90	p__Gemmatimonadetes	f__Gemmatimonadaceae_OTU_90		0.03	0.05	0.04	X	0.18	0.35	0.27	X	0.11	0.20	0.15

79	OTU_8	p__Nitrospirae	g__Nitrospira	X	1.52	2.09	1.80	X	1.08	1.80	1.49	X	0.39	0.96	0.70
80	OTU_10	p__Nitrospirae	g__Nitrospira		0.00	0.01	0.00	X	1.32	2.42	1.99	X	0.66	2.68	1.68
81	OTU_95	p__Nitrospirae	g__Nitrospira	X	0.13	0.20	0.16	X	0.11	0.21	0.16		0.04	0.11	0.06
82	OTU_359	p__Nitrospirae	g__Nitrospira		0.00	0.00	0.00	X	0.22	0.37	0.32	X	0.11	0.41	0.25
83	OTU_9	c__Alphaproteobacteria	g__Bradyrhizobium	X	0.46	0.62	0.52	X	1.80	2.78	2.24	X	0.86	1.90	1.41
84	OTU_21	c__Alphaproteobacteria	c__Alphaproteobacteria_OTU_21		0.00	0.01	0.01		0.00	0.01	0.01	X	0.01	8.66	1.78
85	OTU_75	c__Alphaproteobacteria	g__Devosia		0.04	0.07	0.04	X	0.23	0.27	0.25	X	0.10	0.20	0.15
86	OTU_120	c__Alphaproteobacteria	f__Bradyrhizobiaceae_OTU_120		0.02	0.04	0.03	X	0.20	0.29	0.24	X	0.09	0.31	0.17
87	OTU_35	c__Alphaproteobacteria	f__Hyphomicrobiaceae_OTU_35		0.03	0.05	0.04	X	0.53	0.80	0.66	X	0.30	0.56	0.42
88	OTU_86	c__Alphaproteobacteria	f__Phyllobacteriaceae_OTU_86		0.00	0.01	0.00	X	0.17	0.29	0.22	X	0.09	0.17	0.13
89	OTU_554	c__Alphaproteobacteria	f__Rhodobacteraceae_OTU_554		0.00	0.00	0.00		0.00	0.00	0.00	X	0.00	1.00	0.31
90	OTU_81	c__Alphaproteobacteria	f__Sphingomonadaceae_OTU_81	X	0.10	0.15	0.12	X	0.18	0.30	0.26	X	0.10	0.22	0.16
91	OTU_7	c__Alphaproteobacteria	g__Hyphomicrobium	X	0.39	0.44	0.43	X	2.52	3.12	2.77	X	1.37	2.02	1.68
92	OTU_60	c__Alphaproteobacteria	g__Hyphomicrobium		0.00	0.01	0.01	X	0.22	0.33	0.28	X	0.10	0.23	0.17
93	OTU_109	c__Alphaproteobacteria	g__Hyphomicrobium		0.01	0.03	0.02	X	0.21	0.35	0.27	X	0.11	0.23	0.18
94	OTU_77	c__Alphaproteobacteria	g__Mesorhizobium	X	0.28	0.38	0.34	X	0.54	0.71	0.62	X	0.27	0.58	0.42
95	OTU_43	c__Alphaproteobacteria	g__MNG7	X	0.35	0.42	0.39	X	0.44	0.63	0.54	X	0.32	0.51	0.40
96	OTU_65	c__Alphaproteobacteria	g__Novosphingobium		0.02	0.05	0.03	X	0.08	0.78	0.38	X	0.03	0.48	0.19
97	OTU_143	c__Alphaproteobacteria	o__DB1-14_OTU_143	X	0.15	0.23	0.19		0.00	0.03	0.01		0.00	0.01	0.00
98	OTU_97	c__Alphaproteobacteria	o__DB1-14_OTU_97	X	0.23	0.28	0.26		0.02	0.10	0.05		0.02	0.11	0.04
99	OTU_42	c__Alphaproteobacteria	o__Rhizobiales_OTU_42	X	0.09	0.14	0.11	X	0.22	0.43	0.29	X	0.10	0.40	0.22
100	OTU_4607	c__Alphaproteobacteria	o__Rhizobiales_OTU_4607		0.09	0.13	0.10	X	0.10	0.20	0.15		0.06	0.09	0.08
101	OTU_190	c__Alphaproteobacteria	g__Paracoccus	X	0.14	0.23	0.20		0.06	0.13	0.09		0.02	0.04	0.03
102	OTU_80	c__Alphaproteobacteria	g__Pedomicrobium	X	0.09	0.13	0.11	X	0.26	0.41	0.34	X	0.14	0.22	0.20
103	OTU_4913	c__Alphaproteobacteria	g__Pedomicrobium		0.06	0.09	0.07	X	0.21	0.31	0.26	X	0.13	0.21	0.16
104	OTU_41	c__Alphaproteobacteria	g__Rhodobacter	X	0.45	0.83	0.59	X	0.22	0.37	0.28		0.08	0.25	0.16
105	OTU_58	c__Alphaproteobacteria	g__Rhodobacter	X	0.42	0.55	0.47	X	0.19	0.34	0.27	X	0.11	0.22	0.15
106	OTU_101	c__Alphaproteobacteria	g__Rhodobacter	X	0.13	0.20	0.15	X	0.11	0.19	0.16	X	0.07	0.14	0.10

107	OTU_78	c__Alphaproteobacteria	g__Sphingopyxis	X	0.62	0.77	0.69	X	0.04	0.25	0.13		0.04	0.11	0.07
108	OTU_236	c__Alphaproteobacteria	g__Tabrizicola	X	0.18	0.22	0.20		0.01	0.06	0.03		0.01	0.03	0.02
109	OTU_395	c__Betaproteobacteria	g__188up	X	0.40	0.60	0.53	X	0.09	0.21	0.13	X	0.12	0.18	0.15
110	OTU_2	c__Betaproteobacteria	g__Dechloromonas	X	5.30	6.86	6.22	X	0.30	1.52	0.75	X	0.06	0.19	0.10
111	OTU_22	c__Betaproteobacteria	g__Dechloromonas	X	0.80	1.11	0.95	X	0.56	0.79	0.67	X	0.19	0.34	0.25
112	OTU_40	c__Betaproteobacteria	g__Dechloromonas	X	1.45	2.07	1.66	X	0.04	0.38	0.14	X	0.01	0.09	0.04
113	OTU_118	c__Betaproteobacteria	g__Dechloromonas	X	0.20	0.33	0.27		0.02	0.11	0.05		0.00	0.02	0.01
114	OTU_18	c__Betaproteobacteria	g__Denitratisoma	X	0.83	0.97	0.89	X	0.53	1.30	1.01	X	0.28	0.77	0.52
115	OTU_37	c__Betaproteobacteria	g__Denitratisoma	X	0.34	0.43	0.38	X	0.37	0.64	0.50	X	0.21	0.32	0.26
116	OTU_64	c__Betaproteobacteria	g__Denitratisoma	X	0.26	0.37	0.32	X	0.08	0.21	0.17	X	0.05	0.12	0.09
117	OTU_67	c__Betaproteobacteria	f__A21b_OTU_67	X	0.12	0.21	0.16	X	0.28	0.37	0.32	X	0.11	0.31	0.20
118	OTU_130	c__Betaproteobacteria	f__Comamonadaceae_OTU_130	X	0.19	0.28	0.25	X	0.42	0.47	0.45	X	0.15	0.37	0.25
119	OTU_171	c__Betaproteobacteria	f__Comamonadaceae_OTU_171	X	0.24	0.36	0.31		0.01	0.04	0.03		0.00	0.07	0.02
120	OTU_2062	c__Betaproteobacteria	f__Comamonadaceae_OTU_2062	X	0.15	0.20	0.16		0.00	0.03	0.01		0.00	0.02	0.01
121	OTU_2225	c__Betaproteobacteria	f__Comamonadaceae_OTU_2225		0.02	0.07	0.05		0.00	0.01	0.00	X	0.00	0.73	0.22
122	OTU_3224	c__Betaproteobacteria	f__Comamonadaceae_OTU_3224	X	0.42	0.51	0.48	X	0.24	0.36	0.31	X	0.12	0.33	0.23
123	OTU_3863	c__Betaproteobacteria	f__Comamonadaceae_OTU_3863	X	0.19	0.30	0.24	X	0.27	0.68	0.43	X	0.17	0.47	0.25
124	OTU_44	c__Betaproteobacteria	f__Comamonadaceae_OTU_44	X	0.31	0.69	0.47	X	0.23	0.37	0.30	X	0.06	2.06	0.47
125	OTU_483	c__Betaproteobacteria	f__Comamonadaceae_OTU_483	X	0.06	0.20	0.10		0.04	0.14	0.08		0.05	1.01	0.22
126	OTU_1619	c__Betaproteobacteria	f__RB348_OTU_1619	X	0.09	0.13	0.11	X	0.26	0.45	0.33	X	0.18	0.34	0.24
127	OTU_19	c__Betaproteobacteria	f__RB348_OTU_19	X	0.16	0.27	0.21	X	0.84	1.26	1.10	X	0.46	0.69	0.57
128	OTU_2947	c__Betaproteobacteria	f__Rhodocyclaceae_OTU_2947	X	0.26	0.38	0.32		0.02	0.07	0.04		0.00	0.11	0.03
129	OTU_68	c__Betaproteobacteria	f__Rhodocyclaceae_OTU_68	X	0.05	0.11	0.08	X	0.23	0.53	0.35	X	0.10	0.25	0.15
130	OTU_23	c__Betaproteobacteria	g__Hydrogenophaga	X	0.13	0.20	0.16	X	0.32	0.40	0.36	X	0.19	8.27	2.12
131	OTU_11	c__Betaproteobacteria	g__Limnohabitans		0.00	0.01	0.00		0.00	0.01	0.00	X	0.00	13.34	3.70
132	OTU_2726	c__Betaproteobacteria	g__Malikia		0.02	0.08	0.04		0.03	0.25	0.07	X	0.04	4.81	1.46
133	OTU_105	c__Betaproteobacteria	g__Nitrosomonas	X	0.08	0.10	0.09	X	0.17	0.29	0.21	X	0.06	0.23	0.13
134	OTU_4180	c__Betaproteobacteria	g__Nitrosomonas	X	0.26	0.35	0.31	X	0.09	0.18	0.12		0.02	0.06	0.04

135	OTU_47	c__Betaproteobacteria	o__Burkholderiales_OTU_47	X	0.29	0.34	0.32	X	0.22	0.38	0.29	X	0.10	0.22	0.14
136	OTU_48	c__Betaproteobacteria	g__Propionivibrio	X	0.27	0.52	0.41	X	0.16	0.35	0.22		0.04	0.09	0.08
137	OTU_31	c__Betaproteobacteria	g__Rhodoferax	X	1.72	1.88	1.79	X	0.21	0.64	0.49	X	0.12	0.78	0.48
138	OTU_110	c__Betaproteobacteria	g__Rubrivivax	X	0.29	0.33	0.31	X	0.42	0.56	0.49	X	0.20	0.38	0.28
139	OTU_38	c__Betaproteobacteria	g__Simplicispira	X	0.61	0.86	0.78	X	0.03	0.24	0.09		0.02	0.09	0.04
140	OTU_49	c__Betaproteobacteria	g__spb280	X	0.34	0.41	0.36	X	0.20	0.32	0.25	X	0.12	0.21	0.16
141	OTU_34	c__Betaproteobacteria	g__Sulfuritalea	X	0.20	0.27	0.23	X	0.34	0.98	0.71	X	0.14	0.53	0.34
142	OTU_227	c__Betaproteobacteria	g__Sulfuritalea	X	0.16	0.24	0.19		0.00	0.02	0.01		0.00	0.02	0.00
143	OTU_260	c__Betaproteobacteria	g__Sulfuritalea	X	0.26	0.34	0.30		0.03	0.11	0.06		0.01	0.04	0.02
144	OTU_318	c__Betaproteobacteria	g__Sulfuritalea	X	0.25	0.34	0.30		0.04	0.12	0.08		0.01	0.10	0.04
145	OTU_92	c__Betaproteobacteria	g__Thauera	X	0.13	0.27	0.20	X	0.11	0.24	0.17	X	0.11	0.21	0.16
146	OTU_1411	c__Betaproteobacteria	g__Zoogloea	X	0.24	0.31	0.27	X	0.05	0.11	0.07		0.00	0.08	0.04
147	OTU_147	c__Deltaproteobacteria	g__mle1-27		0.04	0.08	0.06	X	0.12	0.32	0.20	X	0.05	0.25	0.12
148	OTU_73	c__Deltaproteobacteria	g__OM27 clade	X	0.52	0.79	0.63	X	0.01	0.33	0.13	X	0.00	0.09	0.03
149	OTU_83	c__Epsilonproteobacteria	g__Arcobacter	X	0.11	0.21	0.17	X	0.07	0.61	0.19	X	0.02	1.98	0.55
150	OTU_5063	c__Epsilonproteobacteria	g__Arcobacter		0.04	0.07	0.06		0.02	0.17	0.06	X	0.01	0.67	0.18
151	OTU_230	c__Gammaproteobacteria	g__Candidatus Xenovorus	X	0.10	0.21	0.16		0.01	0.06	0.02		0.02	0.05	0.03
152	OTU_113	c__Gammaproteobacteria	f__WD260_OTU_113		0.02	0.05	0.03	X	0.13	0.25	0.19		0.06	0.12	0.08
153	OTU_215	c__Gammaproteobacteria	g__QEEB1BB10	X	0.35	0.45	0.40		0.02	0.05	0.03		0.02	0.07	0.04
154	OTU_159	k__Bacteria_OTU_159	k__Bacteria_OTU_159		0.00	0.00	0.00		0.01	0.15	0.05	X	0.06	0.34	0.16
155	OTU_51	k__Bacteria_OTU_51	k__Bacteria_OTU_51		0.00	0.00	0.00	X	0.34	0.77	0.53	X	0.16	0.30	0.24
156	OTU_71	k__Bacteria_OTU_71	k__Bacteria_OTU_71	X	0.41	0.53	0.47		0.00	0.08	0.02		0.00	0.02	0.00
157	OTU_85	k__Bacteria_OTU_85	k__Bacteria_OTU_85	X	0.29	0.48	0.35		0.01	0.08	0.03		0.00	0.07	0.04
158	OTU_172	k__Unclassified_OTU_172	k__Unclassified_OTU_172		0.04	0.07	0.05	X	0.05	0.16	0.12	X	0.01	0.69	0.22
159	OTU_33	k__Unclassified_OTU_33	k__Unclassified_OTU_33		0.00	0.00	0.00	X	0.54	1.42	0.99	X	0.08	0.53	0.24
160	OTU_98	k__Unclassified_OTU_98	k__Unclassified_OTU_98		0.02	0.05	0.03	X	0.00	0.52	0.17		0.00	0.25	0.08
