# Supplementary material for: Dynamics of the Fouling Layer Microbial Community in a Membrane Bioreactor
Source: PLoS One. 2016 Jul 11;11(7):e0158811. doi: 10.1371/journal.pone.0158811 (PMC4939938; doi:10.1371/journal.pone.0158811)
Supplement: S7 Table — Read abundance of Chloroflexi genera in percentage of all reads in biofilm (BF) and MBR sludge samples. Species-level OTUs are shown. The numbers 1–7 refer to the week of sampling. (PDF) [file pone.0158811.s012.pdf]

**S7 Table: Chloroflexi in biofilm and MBR.** Read abundance of Chloroflexi genera in percentage of all reads in biofilm (BF) and MBR sludge samples. Species-level OTUs are shown. The numbers 1-7 refer to the week of sampling.

| | BF | | | | | | | MBR | | | | | | |
|---|---|---|---|---|---|---|---|---|---|---|---|---|---|---|
| | 1 | 2 | 3 | 4 | 5 | 6 | 7 | 1 | 2 | 3 | 4 | 5 | 6 | 7 |
| p__Chloroflexi | 15.56 | 18.36 | 17.17 | 20.99 | 31.72 | 32.32 | 37.60 | 23.61 | 22.11 | 25.87 | 20.97 | 23.42 | 23.09 | 24.62 |
| g__SBR1029 | 0.23 | 0.42 | 0.20 | 0.35 | 0.50 | 0.44 | 0.45 | 0.54 | 0.59 | 0.47 | 0.61 | 0.58 | 0.57 | 0.66 |
| OTU_250 | 0.08 | 0.06 | 0.04 | 0.10 | 0.08 | 0.12 | 0.10 | 0.04 | 0.07 | 0.04 | 0.04 | 0.07 | 0.05 | 0.08 |
| OTU_3702 | 0.05 | 0.11 | 0.07 | 0.07 | 0.11 | 0.09 | 0.08 | 0.20 | 0.23 | 0.18 | 0.29 | 0.16 | 0.14 | 0.13 |
| OTU_59 | 0.02 | 0.05 | 0.02 | 0.04 | 0.11 | 0.08 | 0.09 | 0.11 | 0.09 | 0.07 | 0.10 | 0.16 | 0.17 | 0.22 |
| g__ | 0.09 | 0.09 | 0.10 | 0.07 | 0.13 | 0.09 | 0.08 | 0.19 | 0.16 | 0.17 | 0.14 | 0.16 | 0.17 | 0.18 |
| OTU_152 | 0.02 | 0.03 | 0.03 | 0.01 | 0.04 | 0.04 | 0.02 | 0.05 | 0.07 | 0.05 | 0.03 | 0.07 | 0.08 | 0.06 |
| g__B45 | 11.65 | 12.69 | 13.34 | 16.48 | 23.86 | 25.16 | 29.27 | 15.56 | 13.98 | 18.35 | 12.88 | 14.97 | 14.74 | 16.74 |
| OTU_1 | 5.19 | 5.57 | 5.73 | 5.34 | 9.08 | 9.13 | 10.66 | 8.63 | 7.44 | 10.17 | 6.24 | 8.52 | 8.22 | 8.95 |
| OTU_117 | 0.38 | 0.57 | 0.41 | 0.58 | 0.74 | 0.75 | 0.81 | 0.66 | 0.70 | 0.77 | 0.63 | 0.55 | 0.53 | 0.63 |
| OTU_1498 | 0.33 | 0.36 | 0.36 | 0.50 | 0.55 | 0.56 | 0.69 | 0.43 | 0.41 | 0.58 | 0.46 | 0.33 | 0.36 | 0.43 |
| OTU_15 | 1.16 | 1.39 | 1.35 | 1.35 | 2.06 | 1.93 | 2.12 | 1.59 | 1.44 | 1.61 | 1.41 | 1.47 | 1.44 | 1.55 |
| OTU_3110 | 2.93 | 2.92 | 3.63 | 5.46 | 6.44 | 7.45 | 9.92 | 2.73 | 2.66 | 3.35 | 2.86 | 2.66 | 2.78 | 3.44 |
| OTU_39 | 0.24 | 0.35 | 0.35 | 0.78 | 1.08 | 1.19 | 1.03 | 0.22 | 0.20 | 0.25 | 0.16 | 0.21 | 0.22 | 0.29 |
| OTU_5102 | 1.05 | 1.06 | 1.04 | 1.52 | 2.80 | 3.05 | 2.66 | 0.80 | 0.73 | 1.01 | 0.62 | 0.80 | 0.82 | 1.01 |
| OTU_55 | 0.30 | 0.36 | 0.37 | 0.81 | 0.94 | 0.89 | 1.17 | 0.34 | 0.27 | 0.42 | 0.35 | 0.32 | 0.23 | 0.33 |
| OTU_939 | 0.04 | 0.10 | 0.08 | 0.10 | 0.13 | 0.12 | 0.15 | 0.15 | 0.13 | 0.17 | 0.13 | 0.12 | 0.11 | 0.10 |
| g__P2CN44 | 0.79 | 1.11 | 0.63 | 0.79 | 1.40 | 1.27 | 1.18 | 1.55 | 1.64 | 1.60 | 1.58 | 1.60 | 1.34 | 1.25 |
| OTU_139 | 0.06 | 0.10 | 0.03 | 0.06 | 0.09 | 0.06 | 0.04 | 0.11 | 0.15 | 0.12 | 0.14 | 0.10 | 0.09 | 0.08 |
| OTU_211 | 0.03 | 0.03 | 0.02 | 0.02 | 0.07 | 0.07 | 0.07 | 0.03 | 0.02 | 0.03 | 0.02 | 0.05 | 0.03 | 0.04 |
| OTU_29 | 0.28 | 0.34 | 0.22 | 0.23 | 0.46 | 0.38 | 0.37 | 0.54 | 0.52 | 0.56 | 0.46 | 0.52 | 0.43 | 0.39 |
| OTU_3030 | 0.01 | 0.03 | 0.02 | 0.04 | 0.11 | 0.11 | 0.09 | 0.01 | 0.04 | 0.02 | 0.05 | 0.04 | 0.05 | 0.03 |

| | | | | | | | | | | | | | | |
|---|---|---|---|---|---|---|---|---|---|---|---|---|---|---|
| OTU_53 | 0.06 | 0.14 | 0.08 | 0.07 | 0.18 | 0.20 | 0.22 | 0.19 | 0.20 | 0.18 | 0.22 | 0.23 | 0.21 | 0.16 |
| OTU_74 | 0.10 | 0.17 | 0.10 | 0.07 | 0.10 | 0.08 | 0.07 | 0.25 | 0.27 | 0.28 | 0.24 | 0.22 | 0.18 | 0.18 |
| g__uncultured | 0.34 | 0.51 | 0.36 | 0.42 | 0.66 | 0.56 | 0.60 | 0.80 | 0.70 | 0.65 | 0.77 | 0.55 | 0.59 | 0.58 |
| OTU_108 | 0.09 | 0.13 | 0.08 | 0.08 | 0.13 | 0.11 | 0.11 | 0.20 | 0.16 | 0.14 | 0.22 | 0.13 | 0.12 | 0.13 |
| OTU_167 | 0.05 | 0.06 | 0.06 | 0.11 | 0.16 | 0.18 | 0.15 | 0.09 | 0.07 | 0.08 | 0.07 | 0.06 | 0.08 | 0.08 |
| g__*Candidatus* Sarcinathrix | 0.07 | 0.16 | 0.09 | 0.10 | 0.14 | 0.17 | 0.16 | 0.20 | 0.17 | 0.17 | 0.25 | 0.19 | 0.19 | 0.16 |
| OTU_82 | 0.04 | 0.06 | 0.03 | 0.04 | 0.10 | 0.07 | 0.08 | 0.08 | 0.06 | 0.04 | 0.11 | 0.10 | 0.09 | 0.07 |
| g__C10_SB1A | 0.08 | 0.15 | 0.12 | 0.14 | 0.78 | 0.90 | 2.59 | 0.11 | 0.12 | 0.14 | 0.20 | 0.79 | 0.78 | 0.70 |
| OTU_14 | 0.03 | 0.07 | 0.05 | 0.06 | 0.48 | 0.59 | 2.12 | 0.04 | 0.04 | 0.06 | 0.08 | 0.55 | 0.58 | 0.50 |
| OTU_1679 | 0.04 | 0.07 | 0.06 | 0.06 | 0.27 | 0.29 | 0.45 | 0.04 | 0.06 | 0.07 | 0.10 | 0.22 | 0.18 | 0.17 |
| g__(AKYG1722) | 0.66 | 1.06 | 0.80 | 0.85 | 1.45 | 1.18 | 0.93 | 1.63 | 1.75 | 1.50 | 1.61 | 1.75 | 1.97 | 1.63 |
| OTU_17 | 0.65 | 1.04 | 0.79 | 0.81 | 1.42 | 1.14 | 0.89 | 1.61 | 1.73 | 1.49 | 1.59 | 1.73 | 1.96 | 1.61 |
| g__(JG30-KF-CM45) | 0.23 | 0.25 | 0.24 | 0.23 | 0.35 | 0.37 | 0.28 | 0.34 | 0.36 | 0.30 | 0.35 | 0.37 | 0.30 | 0.27 |
| OTU_154 | 0.09 | 0.08 | 0.09 | 0.09 | 0.12 | 0.11 | 0.06 | 0.09 | 0.09 | 0.09 | 0.10 | 0.06 | 0.06 | 0.07 |
| g__mle1-48 | 0.20 | 0.33 | 0.23 | 0.26 | 0.47 | 0.39 | 0.29 | 0.54 | 0.65 | 0.51 | 0.56 | 0.49 | 0.55 | 0.59 |
| OTU_62 | 0.10 | 0.20 | 0.12 | 0.16 | 0.28 | 0.21 | 0.13 | 0.32 | 0.38 | 0.30 | 0.31 | 0.23 | 0.29 | 0.36 |
| g__WCHB1-50 | 0.08 | 0.09 | 0.11 | 0.10 | 0.23 | 0.22 | 0.09 | 0.22 | 0.18 | 0.23 | 0.18 | 0.30 | 0.22 | 0.19 |
| OTU_140 | 0.08 | 0.09 | 0.11 | 0.10 | 0.22 | 0.22 | 0.08 | 0.21 | 0.18 | 0.22 | 0.16 | 0.29 | 0.22 | 0.19 |
