# Supplementary material for: Dynamics of the Fouling Layer Microbial Community in a Membrane Bioreactor
Source: PLoS One. 2016 Jul 11;11(7):e0158811. doi: 10.1371/journal.pone.0158811 (PMC4939938; doi:10.1371/journal.pone.0158811)
Supplement: S8 Table — Read abundance of common AOBs (genus Nitrosomonas) and NOBs (genera Nitrospira and Ca. Nitrotoga) in percentage of all reads in CAS and MBR sludge samples. Species-level OTUs are shown (97% sequence similarity cut-off). The numbers 1–7 refer to the week of sampling. (PDF) [file pone.0158811.s013.pdf]

S8 Table: **AOBs and NOBs in CAS and MBR.** Read abundance of common AOBs (genus *Nitrosomonas*) and NOBs (genera *Nitrospira* and *Ca.* Nitrotoga) in percentage of all reads in CAS and MBR sludge samples. Species-level OTUs are shown (97% sequence similarity cut-off). The numbers 1-7 refer to the week of sampling.

	CAS							MBR						
	1	2	3	4	5	6	7	1	2	3	4	5	6	7
AOBs														
g__*Nitrosomonas*	0.74	0.78	0.62	0.73	0.68	0.67	0.67	0.67	0.53	0.40	0.48	0.61	0.46	0.39
OTU_105	0.10	0.10	0.08	0.08	0.10	0.10	0.08	0.29	0.22	0.18	0.20	0.21	0.17	0.18
OTU_299	0.12	0.15	0.13	0.16	0.13	0.11	0.16	0.07	0.04	0.03	0.03	0.05	0.05	0.03
OTU_4180	0.35	0.35	0.26	0.33	0.28	0.30	0.27	0.15	0.12	0.10	0.12	0.18	0.09	0.09
NOBs	0.00	0.00	0.00	0.00	0.00	0.00	0.00	0.00	0.00	0.00	0.00	0.00	0.00	0.00
g__*Nitrospira*	1.96	2.02	2.30	1.79	1.69	2.16	2.05	3.63	3.67	4.17	4.23	4.37	4.20	3.58
s__sublineageI	1.78	1.84	2.14	1.63	1.54	1.95	1.92	1.82	1.62	1.71	1.49	1.49	1.33	1.10
OTU_8	1.75	1.82	2.09	1.59	1.52	1.92	1.88	1.80	1.60	1.68	1.48	1.48	1.32	1.08
s__sublineageII	0.17	0.18	0.16	0.16	0.15	0.20	0.13	1.81	2.05	2.47	2.74	2.88	2.87	2.49
OTU_10	0.00	0.00	0.01	0.00	0.00	0.00	0.00	1.32	1.50	2.00	2.21	2.33	2.42	2.15
OTU_359	0.00	0.00	0.00	0.00	0.00	0.00	0.00	0.30	0.34	0.31	0.37	0.35	0.32	0.22
OTU_95	0.17	0.18	0.15	0.16	0.15	0.20	0.13	0.19	0.21	0.15	0.16	0.20	0.13	0.11
g__*Candidatus* Nitrotoga	0.13	0.18	0.14	0.15	0.10	0.07	0.11	0.00	0.00	0.00	0.00	0.04	0.02	0.02
OTU_205	0.13	0.18	0.14	0.15	0.10	0.07	0.11	0.00	0.00	0.00	0.00	0.04	0.02	0.02
