# Supplementary material for: Dynamics of the Fouling Layer Microbial Community in a Membrane Bioreactor
Source: PLoS One. 2016 Jul 11;11(7):e0158811. doi: 10.1371/journal.pone.0158811 (PMC4939938; doi:10.1371/journal.pone.0158811)
Supplement: S9 Table — Read abundance of common PAOs (genus Ca. Accumulibacter and Tetrasphaera) in percentage of all reads in CAS and MBR sludge samples. Species-level OTUs (97% sequence similarity cut-off) are shown. The numbers 1–7 refer to the week of sampling. (PDF) [file pone.0158811.s014.pdf]

S9 Table: Bacteria involved in P-removal in CAS and MBR. Read abundance of common PAOs (genus *Ca.* Accumulibacter and *Tetrasphaera*) in percentage of all reads in CAS and MBR sludge samples. Species-level OTUs (97 % sequence similarity cut-off) are shown. The numbers 1-7 refer to the week of sampling.

	CAS							MBR						
	1	2	3	4	5	6	7	1	2	3	4	5	6	7
PAOs														
g__*Candidatus* Accumulibacter	0.51	0.54	0.45	0.45	0.42	0.52	0.45	0.06	0.08	0.07	0.08	0.15	0.10	0.11
OTU_288	0.12	0.16	0.13	0.18	0.14	0.14	0.14	0.03	0.04	0.04	0.04	0.06	0.04	0.04
OTU_992	0.14	0.13	0.12	0.11	0.13	0.15	0.14	0.01	0.01	0.01	0.01	0.05	0.02	0.03
g__*Tetrasphaera*	2.62	3.06	2.72	2.62	4.06	3.26	3.18	0.28	0.32	0.29	0.23	1.34	1.16	0.98
OTU_163	0.13	0.16	0.15	0.13	0.24	0.13	0.19	0.00	0.00	0.00	0.01	0.05	0.07	0.03
OTU_24	2.33	2.68	2.39	2.32	3.56	2.90	2.82	0.21	0.27	0.22	0.18	1.17	0.95	0.85
